# Predictors of death after receiving a modified Blalock-Taussig shunt in cyanotic heart children: A competing risk analysis

**DOI:** 10.1371/journal.pone.0245754

**Published:** 2021-01-22

**Authors:** Maliwan Oofuvong, Jutarat Tanasansuttiporn, Wirat Wasinwong, Voravit Chittithavorn, Pongsanae Duangpakdee, Jirayut Jarutach, Qistina Yunuswangsa

**Affiliations:** 1 Department of Anesthesiology, Faculty of Medicine, Prince of Songkla University, Songkhla, Thailand; 2 Division of Cardiothoracic Surgery, Department of Surgery, Faculty of Medicine, Prince of Songkla University, Songkhla, Thailand; 3 Division of Cardiology, Department of Pediatrics, Faculty of Medicine, Prince of Songkla University, Songkhla, Thailand; Ohio State University Wexner Medical Center Department of Surgery, UNITED STATES

## Abstract

**Objective:**

To determine risk factors affecting time-to-death ≤90 and >90 days in children who underwent a modified Blalock-Taussig shunt (MBTS).

**Methods:**

Data from a retrospective cohort study were obtained from children aged 0–3 years who experienced MBTS between 2005 and 2016. Time-to-death (prior to Glenn/repair), time-to-alive up until December 2017 without repair, and time-to-progression to Glenn/repair following MBTS were presented using competing risks survival analysis. Demographic, surgical and anesthesia-related factors were recorded. Time-to-death ≤90 days and >90 days was analyzed using multivariate time-dependent Cox regression models to identify independent predictors and presented by adjusted hazard ratios (HR) and 95% confidence intervals (CI).

**Results:**

Of 380 children, 119 died, 122 survived and 139 progressed to Glenn/repair. Time-to-death probability (95% CI) within 90 days was 0.18 (0.14–0.22). Predictors of time-to-death ≤90 days (n = 63) were low weight (<3 kg) (HR 7.6, 95% CI:2.8–20.4), preoperative ventilator support (HR 2.7, 95% CI:1.3–5.6), postoperative shunt thrombosis (HR 5.0, 95% CI:2.4–10.4), bleeding (HR 4.5, 95% CI:2.1–9.4) and renal failure (HR 4.1, 95% CI:1.5–10.9). Predictors of time-to-death >90 days (n = 56) were children diagnosed with pulmonary atresia with ventricular septal defect and single ventricle (compared to tetralogy of fallot) (HR 3.2, 95% CI:1.2–7.7 and HR 3.1, 95% CI:1.3–7.6, respectively), shunt size/weight ratio >1.1 vs <0.65 (HR 6.8, 95% CI:1.4–32.6) and longer duration of mechanical ventilator (HR 1.002, 95% CI:1.001–1.004). Shunt size/weight ratio ≥1.0 (vs <1.0) and ≥0.65 (vs <0.65) were predictors for overall time-to-death in neonates and toddlers, respectively (HR 13.1, 95% CI:2.8–61.4 and HR 7.8, 95% CI:1.7–34.8, respectively).

**Conclusions:**

Perioperative factors were associated with time-to-death ≤90 days, whereas particular cardiac defect, larger shunt size/weight ratio, and longer mechanical ventilation were associated with time-to-death >90 days after receiving MBTS. Larger shunt size/weight ratio should be reevaluated within 90 days to minimize the risk of shunt over flow.

## Introduction

Systemic to pulmonary artery shunt became a palliative procedure for children with complex congenital heart disease to help increase blood flow to the lung and to reduce cyanosis in children with cyanotic heart disease [1]. Currently, a modified Blalock-Taussig shunt (MBTS) is the most common palliative shunt. Since 2000, the discharge mortality rate after MBTS has ranged between 3.7 and 14% [1–5].

Previous studies reported many factors associated with in-hospital mortality and the need for complementary support such as postoperative low cardiac output, unplanned reoperation and/or extracorporeal membrane oxygenation after receiving MBTS [5]. The preoperative factors included age [4], low body weight at the time of surgery [3–5], underlying cardiac diagnosis (pulmonary atresia with intact ventricular septal and Ebstein’s anomaly [5, 6], preoperative mechanical ventilation support and metabolic acidosis [5]. The most common complication that led to postoperative mortality was shunt thrombosis [7]. However, most studies were performed in the neonate group [5, 7, 8] and few studies were carried out in all age groups [4, 6]. Moreover, reports on predictors of time-to-death after receiving MBTS are scarce. Thus, this study aims to determine risk factors affecting time-to-death ≤90 days and >90 days compared to children waiting for or progressed to the next stage (Glenn or biventricular repair) after receiving MBTS.

## Materials and methods

This was a historical cohort study. The study was approved by the Institutional Ethics Committee of the Faculty of Medicine, Prince of Songkla University, Songkhla, Thailand on December 2016 (REC 59-301-08-01). After the study was approved, all electronic records of consecutive patients aged 0–3 years admitted for MBTS between January 2005 and December 2016 were reviewed. The source of medical records and anesthetic records were from the hospital information system of Songklanagarind Hospital. All data were fully anonymized before accessed by the investigators. Children lost to follow up were excluded. The DOI link by Protocols.io is dx.doi.org/10.17504/protocols.io.bqz8mx9w.

### Standard operating procedures

In the intraoperative period the anesthetic technique was designed by the anesthesiologist in charge and was similar for all children. Anesthesia was induced with fentanyl, ketamine and/or propofol for blunt airway reflex. Cisatracurium was used for the intubation and maintenance periods. Fraction of inspired oxygen between 0.6–1 was used to restore oxygen saturation (SpO_2_) greater than baseline SpO_2_. Heparin was administered in a dose of 100 units per kilogram in all children before shunt placement. Shunt size selection was based on the basis of body weight, size of pulmonary artery to be shunted and surgeon preference. After the operation, inotropic support (norepinephrine or epinephrine 0.05–0.1 mcg/kg/min) was commenced intraoperatively and continued through the postoperative period to maintain adequate blood pressure to promote shunt flow. All patients were ventilated postoperatively and transferred to the pediatric intensive care unit (PICU). If there was no evidence of bleeding, heparin was continuously infused for 48 h, followed by a daily oral dose of aspirin.

### Predictors and potential confounding variables

The variables collected were categorized into preoperative (patient and anesthesia-related factors), intraoperative (surgery and anesthesia-related factors) and postoperative factors. Patient-related factors included age, weight at surgery, premature birth (gestational age <37 weeks), cardiac diagnosis (categorized as single ventricle, pulmonary atresia with ventricular septal defect (PA-VSD), tetralogy of fallot (TOF) and others), presentation of noncardiac abnormality (categorized as a syndrome such as heterotaxy, dextrocardia, or chronic lung disease), history of receiving a previous MBTS and preoperative inotropic drug use and ventilator support. The cardiac diagnoses of a single ventricle were pulmonary atresia, pulmonary atresia with intact ventricular septum (PAIVS), tricuspid atresia, double inlet left ventricle, double outlet right ventricle, and complete atrioventricular canal defect with pulmonary atresia. Other complex cardiac diseases included transposition of great arteries (TGA), total anomalous pulmonary venous return (TAPVR) and hypoplastic left heart syndrome. Anesthesia-related factors included American Society of Anesthesiologists (ASA) classification (2–4), intraoperative inotropic drug use, intraoperative cardiac failure (requiring at least 2 inotropic drugs), intraoperative hemodilution (requiring phlebotomy to keep hematocrit <60% before surgery), intraoperative hypoxemia (SpO_2_ <75%), intraoperative hypoxic spell (acute hypoxemia with response to fluid and sodium bicarbonate administration) and bradycardia (heart rate below baseline by child’s age with required atropine). Surgery-related factors included shunt size, duration, postoperative SpO_2_, duration of postoperative mechanical ventilator use, length of intensive care unit (ICU) stay, length of hospital stay, postoperative complications (shunt thrombosis, bleeding, pneumothorax, perigraft seroma, chylothorax, sepsis, renal failure), required shunt revision and reoperative thoracotomy during admission and readmission within 30 days. Shunt thrombosis was defined as loss of shunt murmur combined with no shunt flow diagnosed by echocardiography. Pneumothorax was defined as abnormal collection of air into the pleural space diagnosed by chest X-ray or computed tomography (CT) scan. Perigraft seroma was defined as collection around prosthesis diagnosed by a pediatric cardiologist. Chylothorax was defined as the presence of chyle in the pleural spaces. Sepsis was diagnosed as presumed or proven infection with systemic inflammation. Renal failure was diagnosed as a doubling of baseline serum creatinine level as recorded on admission to PICU. Total number of MBTS operations and other surgeries related to MBTS was defined as the total number of MBTS operations that the patient received previously and currently including shunt revision and reoperative thoracotomy related to complications (hemothorax, chylothorax, diaphragmatic plication) after MBTS in all admissions.

### Outcomes of interest

Outcomes of interest were time-to-death defined as the time from postoperative MBTS to death ≤90 days and >90 days. Censored was defined as the time of postoperative MBTS to time of progression to the next stage or when data collection ended (December 31, 2017) if the child was still alive without progression to the next stage of repair. The next stages of repair were biventricular repair or Glenn’s operation for univentricular heart.

### Sample size calculation

We estimated that the overall mortality rate in children receiving MBTS was 25%. For survival analysis, a hazard ratio (r) for time-to-death of 1.5, which represented a clinically significant increase in hazard, was used to calculate the required sample size under a significance level of 0.05 and 80% power using the following formula [9, 10]:

$$n={\left(Z_{\alpha /2}+Z_{\beta }\right)}^{2}\times \frac{(1+1/m)/p}{\ln({r)}^{2}}$$

where *p* = 1- *p*_a_ (exp(-*ln*(2)*F*/m)), *p*_a_ = {1- exp(-*ln*(2)*A*/m)}/*ln*(2)*A*/m, *F* is the additional follow-up time after the end of recruitment = 325 days, *A* is the accrual time during which subjects are recruited into the study = 150 days, and *m* is the ratio of number of unexposed to certain risk factors per exposed subject = 2. With these parameters, the required sample size was 96 children for exposed cases and 192 children for unexposed cases. The total sample size was increased to 360 to account for a 20% dropout rate.

### Statistical analysis

Data record forms were created and information was abstracted from the electronic medical records, then double-entered into a database using EpiData version 3.1. R software was used to analyze the data (R version 4.0.2, R Core Team, Vienna). Continuous variables were presented using the median and interquartile range for non-normally distributed data or mean and standard deviation for normally distributed data. Categorical variables were compared using Fisher's exact test or Pearson’s Chi-square test. Continuous variables were compared using the Kruskal-Wallis tests if more than two groups were compared. Post-hoc analysis was performed for multiple comparisons between groups when the overall differences were significant.

Time-to-death (prior to the next stage), time to progression to the next stage (Glenn/repair) and event-free survival time without undergoing Glenn/repair were performed by survival analysis using competing risk analysis [11]. For predictor analysis, time to progression to Glenn/repair was combined with time to alive without Glenn/repair as the censors. Time-to-death prior to Glenn repair (≤90 days and >90 days) was performed using time dependent covariates and time dependent coefficients in the Cox model [12]. Time in (0, 91), time out (90, censored) and new status (0 = alive/Glenn, 1 = death) were created by expanding the data into time to alive/Glenn or death between 0–90 days and from 91 days to censored. The potential risk factors of time-to-death (≤90 days and >90 days) were included in the multivariate time dependent Cox model if the p-value was ≤0.1 in the univariate analysis. The association between time-to-death (≤90 days and >90 days) and potential predictors were presented as adjusted hazard ratios and 95% confidence intervals and considered statistically significant if the p-value was <0.05. A subgroup analysis among children aged ≤1 month and >1 month under the time dependent Cox model was also performed to examine the associated predictors in the same manner as the main outcomes.

## Results

**Fig 1:**
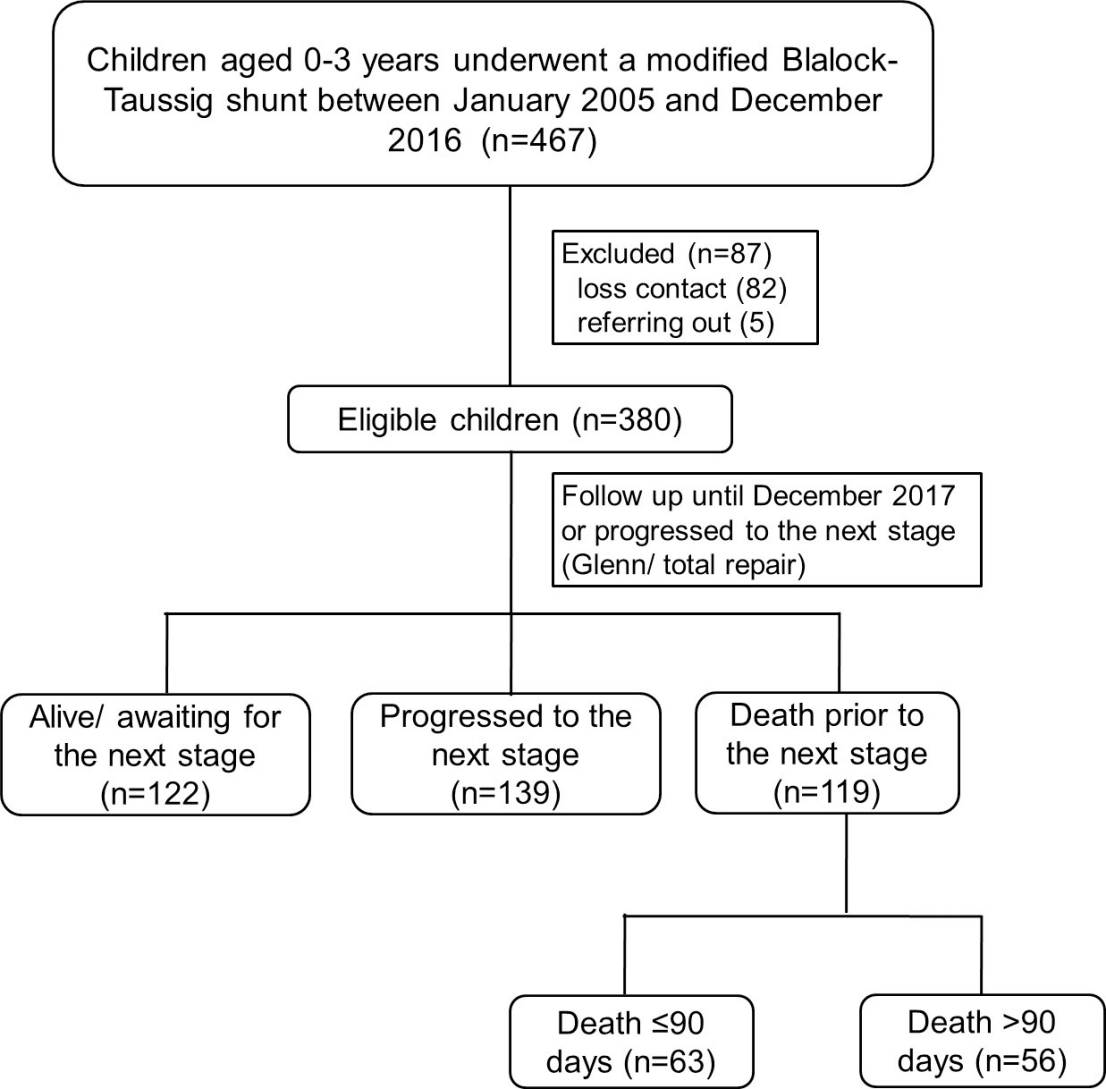
Flow diagram of the study. GA, general anesthesia.

A total of 467 children underwent MBTS between January 2005 and December 2016. We excluded 87 children because of loss of contact (n = 82) and referral to hometown hospital (n = 5). Finally, a total of 380 children were recruited (Fig 1), of which 59 (15.5%) died during admission. Among the 321 children who were discharged, 139 (36.6%) progressed to the next stage (Glenn shunt or total repair, 2 children at days 19 and 43, and 137 children after 90 days) and 60 children (15.8%) died prior to the next stage (4 children died within 90 days and 56 children died after 90 days). A total of 142 single ventricle children had PAIVS (35.5%), tricuspid atresia (24.6%), double outlet right ventricle with pulmonary atresia (22.5%), complete atrioventricular canal defect with pulmonary atresia (16.2%), and double inlet left ventricle (2.1%).

### Competing risk analysis of outcomes after MBTS

**Fig 2:**
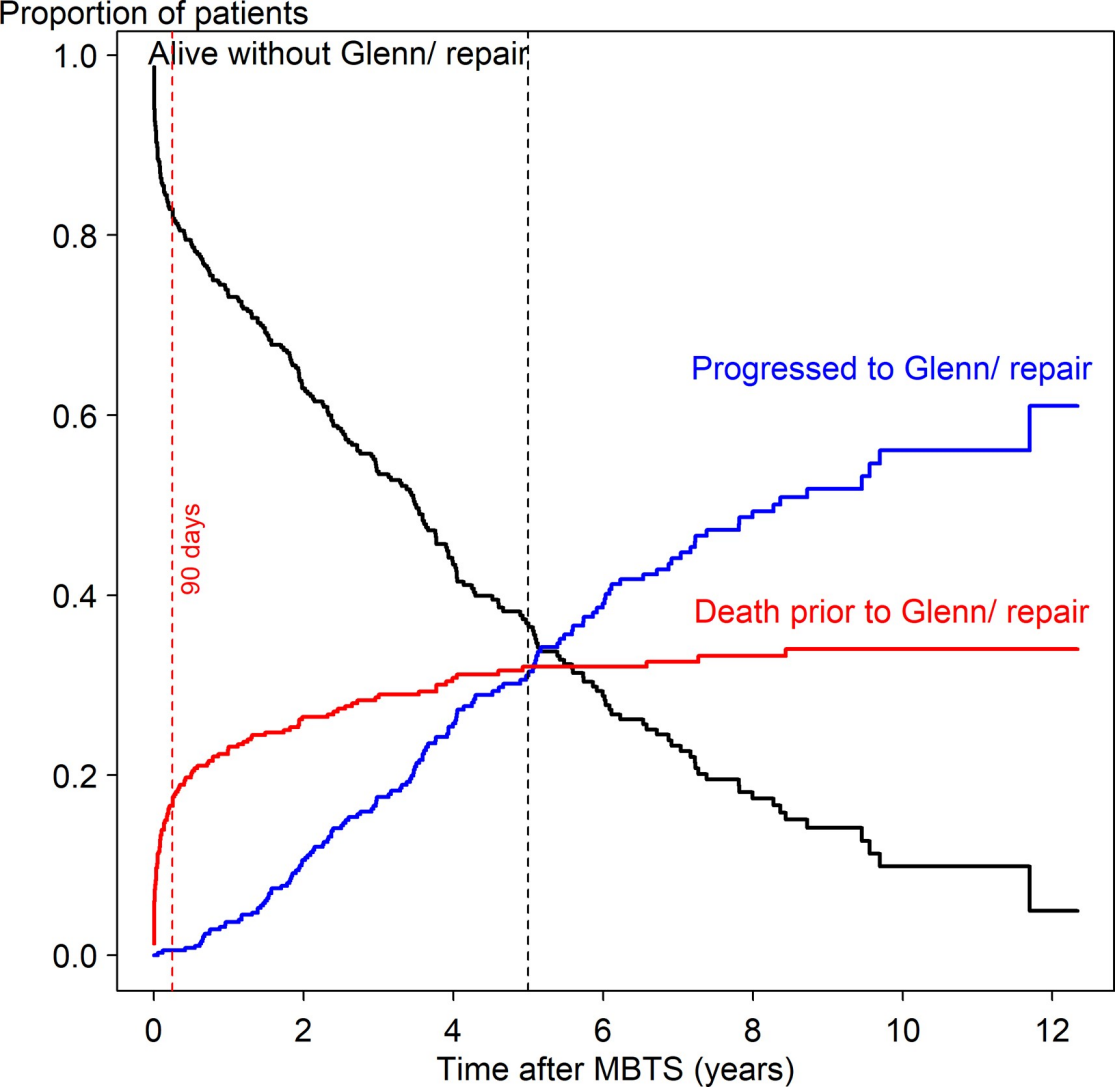
Competing risk survival analysis of time-to-death (prior to Glenn/repair), time-to-survival without Glenn/repair and time-to-progression to Glenn/repair following a modified Blalock-Taussig shunt.

Fig 2 shows competing risks of events among deaths, surviving children without repair and children who progressed to the next stage following MBTS. The proportion of children who underwent Glenn/repair increased at around 5 months and peaked at 6.5 years after MBTS. The curve of death prior the next stage was steepest at 3 months (90 days), while the upward convexity at 1.5 years indicated a lowering of the death rate over this period. Competing risk analysis shows that at 5 years after MBTS, 32% had died and only 31.5% had undergone Glenn/repair. The overall time-to-death probability (95% CI) within 90 days was 0.18 (0.14–0.22).

#### Survival analysis of time-to-death ≤90 and >90 days after MBTS

Time to censored was either time after MBTS to the next stage (Glenn/repair) or time to survival up until December 2017. Data was expanded into time-to-death ≤90 days (n = 380, death 63 children) and time-to death >90 days (n = 315, death 56 children). S1A Fig shows the Kaplan-Meier curve of the time-to-death ≤90 days after receiving MBTS. The probability of survival at 75 days was 0.83 (0.80, 0.87). S1B Fig shows the Nelson Aalen cumulative hazard plot of the event of death after receiving MBTS. The curve of death over time was steepest at 1 day, while the upward convexity at 30 days indicated a lowering of the death rate over this period. S2A Fig shows the Kaplan-Meier curve of the time-to-death >90 days after receiving MBTS. The probability of survival at 3,082 days was 0.70 (0.61, 0.81). S2B Fig shows the Nelson Aalen cumulative hazard plot of the event of death after receiving MBTS. The curve of death over time was steepest at 180 days, while the upward convexity at 500 days indicated a lowering of the death rate over this period.

### Preoperative, intraoperative and postoperative factors related with death

Comparison of preoperative, intraoperative and postoperative factors between the three groups including alive/repair, death ≤90 days, death *>*90 days among children who underwent MBTS are shown in Tables 1 and 2. Age < 1 month, weight <3 kg, dextrocardia, preoperative ventilation support, diagnosed with single ventricle, preoperative PGE_1_ and inotrope use, ASA classification and emergency surgery were significantly associated with death ≤90 and > 90 days. Median shunt size/weight ratio in children who died ≤90 days and >90 days were significantly higher than children who survived (1.1, 1.2, and 0.7, p <0.001, respectively). S3 Fig shows the Kaplan-Meier survival curve of shunt size/weight ratio among children who died ≤90 days. The two optimal cut points for shunt size/weight ratio and time-to-death ≤90 days were 0.65 and 1.1. S4 Fig shows the Kaplan-Meier survival curve of shunt size/weight ratio among children who died >90 days. The two optimal cut points for shunt size/weight ratio and time-to-death >90 days were 0.7 and 1.1. Therefore, shunt size/weight ratio was divided into <0.65, 0.65–1.1 and >1.1 (Table 2). The median hours of mechanical ventilation in children who died ≤90 days and >90 days were significantly higher than children who survived (44, 54 and 27 hours, p <0.001, respectively)

**Table 1:** The demographic data, surgery and anesthesia factors related to postoperative death (n = 380).

Variable	Alive (n = 261)	Dead >90 days (n = 56)	Dead ≤90 days (n = 63)	p value
Age (months), median [IQR]	7.0 [0.8, 22.8]	0.6 [0.2, 4.3]	1.1 [0.3, 4.6]	<0.001 ^a^^b^
< 1	78 (29.9)	34 (60.7)	30 (47.6)	<0.001
1–12	71 (27.2)	13 (23.2)	25 (39.7)	
> 12	112 (42.9)	9 (16.1)	8 (12.7)	
Weight (kg), median [IQR]	6.4 [3.2, 9.4]	3.3 [2.7, 4.9]	3.3 [2.7, 5.2]	<0.001 ^a^^b^
Weight <3 kg	45 (17.2)	25 (44.6)	25 (39.7)	<0.001
History of prematurity	35 (13.4)	7 (12.5)	16 (25.4)	0.049 ^b^
Heterotaxy syndrome	16 (6.1)	5 (8.9)	8 (12.7)	0.180
Others syndrome	24 (9.2)	2 (3.6)	3 (4.8)	0.304
Dextrocardia	14 (5.4)	7 (12.5)	9 (14.3)	0.023 ^b^
Chronic lung disease	31 (11.9)	8 (14.3)	10 (15.9)	0.659
Sepsis	12 (4.6)	1 (1.8)	6 (9.5)	0.164
Hypoxic spell	4 (1.5)	2 (3.6)	4 (6.3)	0.059
Congestive heart failure	7 (2.7)	2 (3.6)	5 (7.9)	0.111
Ventilator support	49 (18.8)	17 (30.4)	34 (54.0)	<0.001 ^b^^c^
Diagnosis				<0.001^1^^a^^b^^,^^2^^a^^,^^3^^b^
Tetralogy of fallot (ref)	106 (40.6)	8 (14.3)	11 (17.5)	
Single ventricle	81 (31.0)	28 (50.0)	33 (52.4)	
PA-VSD	54 (20.7)	18 (32.1)	8 (12.7)	
Complex heart disease	20 (7.7)	2 (3.6)	11 (17.5)	
SpO_2_ (%), median [IQR]	79 [72, 86]	83 [78, 87]	82 [76, 88]	0.015 ^a^
PGE_1_ use	68 (26.1)	34 (60.7)	32 (50.8)	<0.001 ^a^^b^
Number of MBTS operations				0.107
One	216 (82.8)	53 (94.6)	58 (92.1)	
Two	41 (15.7)	3 (5.4)	5 (7.9)	
Three or more	4 (1.5)	0 (0)	0 (0)	
Inotropic drugs used	36 (13.8)	12 (21.4)	21 (33.3)	0.001 ^b^
Adrenaline	0 (0)	0 (0)	2 (3.2)	0.049 ^b^
Noradrenaline	29 (11.1)	6 (10.7)	13 (20.6)	0.111
Dopamine	8 (3.1)	5 (8.9)	12 (19.0)	<0.001 ^b^
Dobutamine	2 (0.8)	1 (1.8)	2 (3.2)	0.180
ASA classification				0.009 ^4^^b^
2	7 (2.7)	0 (0)	0 (0)	
3 (ref)	213 (81.6)	43 (76.8)	41 (65.1)	
4	41 (15.7)	13 (23.2)	22 (34.9)	
Emergency surgery	155 (59.4)	44 (78.6)	52 (82.5)	<0.001 ^a^^b^

Data presented as frequency (%) unless stated otherwise. ASA, American Society of Anesthesiologists; IQR, interquartile range; MBTS, modified Blalock-Taussig shunt; PA-VSD, pulmonary atresia with ventricular septal defect; PGE_1_, prostaglandin E1; SpO_2_, oxygen saturation.

For multiple comparison

^a^ p<0.05 between Alive vs. Dead >90 days

^b^ p<0.05 between Alive vs. Dead ≤90 days

^c^ p<0.05 between Dead >90 days vs. Dead ≤90 days, and for multilevel variables, only p-values <0.001 are reported. Ref: reference group.

^1^Single ventricle vs Tetralogy of fallot.

^2^PA-VSD vs Tetralogy of fallot.

^3^Complex heart disease vs Tetralogy of fallot.

^4^ASA 4 vs ASA 3.

**Table 2:** Intraoperative surgery and anesthesia factors related to postoperative death (n = 380).

Variable	Alive (n = 261)	Dead >90 days (n = 56)	Dead ≤90 days (n = 63)	p value
**Intraoperative period**				
Inotropic drug use	194 (74.3)	40 (71.4)	51 (81.0)	0.442
Adrenaline	3 (1.1)	0 (0)	6 (9.5)	0.002 ^c^
Noradrenaline	110 (42.1)	21 (37.5)	31 (49.2)	0.419
Dopamine	119 (45.6)	27 (48.2)	33 (52.4)	0.615
Dobutamine	3 (1.1)	1 (1.8)	1 (1.6)	0.650
Intraoperative hemodilution	19 (7.3)	2 (3.6)	2 (3.2)	0.478
Shunt size (mm. ID), median [IQR]	4.0 [4.0, 5.0]	4.0 [3.4, 4.0]	3.5 [3.5, 4.0]	<0.001 ^a^^b^
Shunt size/weight ratio, median [IQR]	0.7 [0.5, 1.1]	1.2 [0.8, 1.3]	1.1 [0.7, 1.3]	<0.001 ^a^^b^
<0.65	129 (49.4)	6 (10.7)	13 (20.6)	<0.001
0.65–1.1	81 (31.0)	25 (44.6)	26 (41.3)	
>1.1	51 (19.5)	25 (44.6)	24 (38.1)	
Intraoperative adverse events				
Bradycardia	17 (6.5)	7 (12.5)	9 (14.3)	0.079
Hypoxemia	3 (1.1)	4 (7.1)	6 (9.5)	<0.001 ^a^^b^
Hypoxic spell	62 (23.8)	18 (32.1)	30 (47.6)	0.001 ^b^
Cardiac failure	0 (0)	0 (0)	5 (7.9)	<0.001 ^b^
Cardiac arrest	1 (0.4)	0 (0)	7 (11.1)	<0.001 ^b^^c^
Blood loss (ml), median [IQR]	10 [5, 20]	10 [5, 16.2]	10 [5, 20]	0.193
Duration of surgery (minutes), median [IQR]	135 [120, 155]	130 [105, 150]	135 [120,165]	0.214
**Postoperative period**				
SpO_2_ (%), median [IQR]	93 [89, 97]	95 [87, 98]	91 [83, 96]	0.094
Duration of mechanical ventilator (hours), median [IQR]	27 [16, 70]	54 [35, 150]	44 [18, 130]	<0.001 ^a^
Length of ICU stay (days), median [IQR]	3 [2, 8]	7 [4, 16]	4 [1, 13]	<0.001 ^a^^c^
Length of hospital stay (days), median [IQR]	13 [9, 20]	15 [10, 30]	12 [6, 32]	0.267
Postoperative complication				
Shunt thrombosis	16 (6.1)	6 (10.7)	22 (34.9)	<0.001 ^b^^c^
Bleeding	10 (3.8)	3 (5.4)	20 (31.7)	<0.001 ^b^^c^
Pneumothorax	18 (6.9)	10 (17.9)	9 (14.3)	0.018 ^a^
Perigraft seroma/chylothorax	24 (9.2)	3 (5.4)	1 (1.6)	0.093
Pneumonia	58 (22.2)	17 (30.4)	21 (33.3)	0.121
Sepsis	17 (6.5)	10 (17.9)	26 (41.3)	<0.001 ^a^^b^^c^
Renal failure	1 (0.4)	0 (0)	9 (14.3)	<0.001 ^b^^c^
Shunt revision	15 (5.7)	8 (14.3)	12 (19.0)	0.002 ^a^^b^
Number of reoperative thoracotomies during admission, median [IQR]	0 [0, 0]	0 [0, 1]	0 [0, 1]	<0.001 ^b^
Readmission within 30 days				0.010 ^1^ ^a^^b^
No (ref)	222 (85.1)	40 (71.7)	44 (69.8)	
Yes	2 (0.8)	1 (1.8)	2 (3.2)	
Still admitted	37 (14.2)	15 (26.8)	17 (27.0)	
Admission >90 days	4 (1.5)	3 (5.4)	0 (0)	0.095
Number of MBTS operations and other surgeries related to MBTS in all admissions, median [IQR]	2 [1, 2]	1 [1, 2]	1 [1, 2]	0.803

Data presented as frequency (%) unless stated otherwise.

ICU, intensive care unit; IQR, interquartile range; ID, internal diameter; MBTS, modified Blalock-Taussig shunt.

For multiple comparison

^a^ p<0.05 between Alive vs. Dead >90 days

^b^ p<0.05 between Alive vs. Dead ≤90 days

^c^ p<0.05 between Dead >90 days vs. Dead ≤90 days, Ref: reference group.

^1^Still admitted vs not readmitted.

Intraoperative adverse events (hypoxemia, hypoxic spell, cardiac failure and cardiac arrest) and postoperative complications (shunt thrombosis, bleeding, renal failure and sepsis) in children who died ≤90 days and >90 days were significantly higher than children who survived. Shunt revision arose from shunt occlusion or shunt thrombosis mostly performed in the opposite site with the same (85%) or higher (15%) shunt size. Reoperative thoracotomy at the same admission mostly arose from bleeding, perigraft seroma and diaphragm paralysis.

### Risk factors of time-to-death ≤90 and >90 days by time dependent Cox model after MBTS

**Fig 3:**
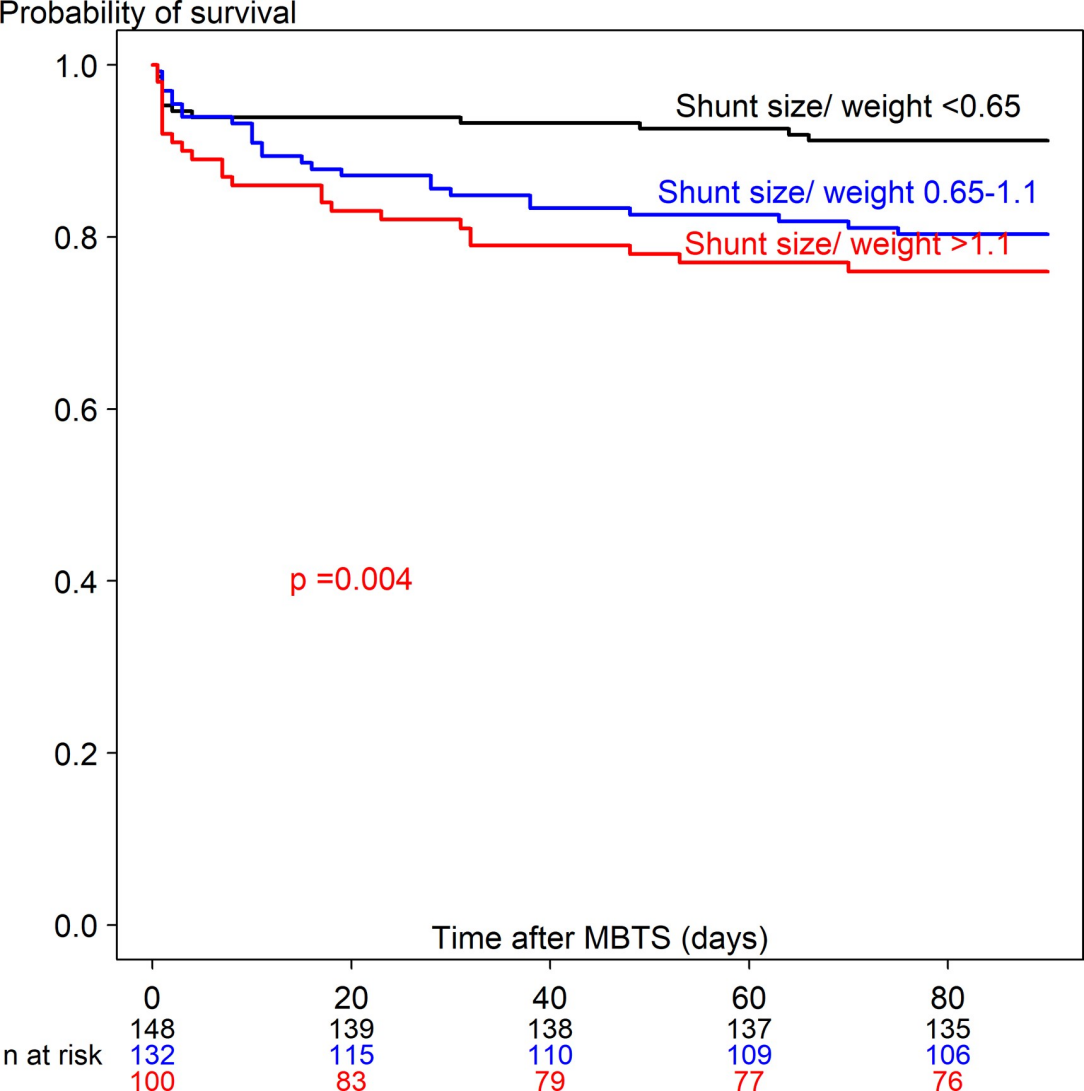
Kaplan-Meier curve of time-to-death ≤90 days after receiving a modified Blalock-Taussig shunt among three shunt size/weight ratios.

**Fig 4:**
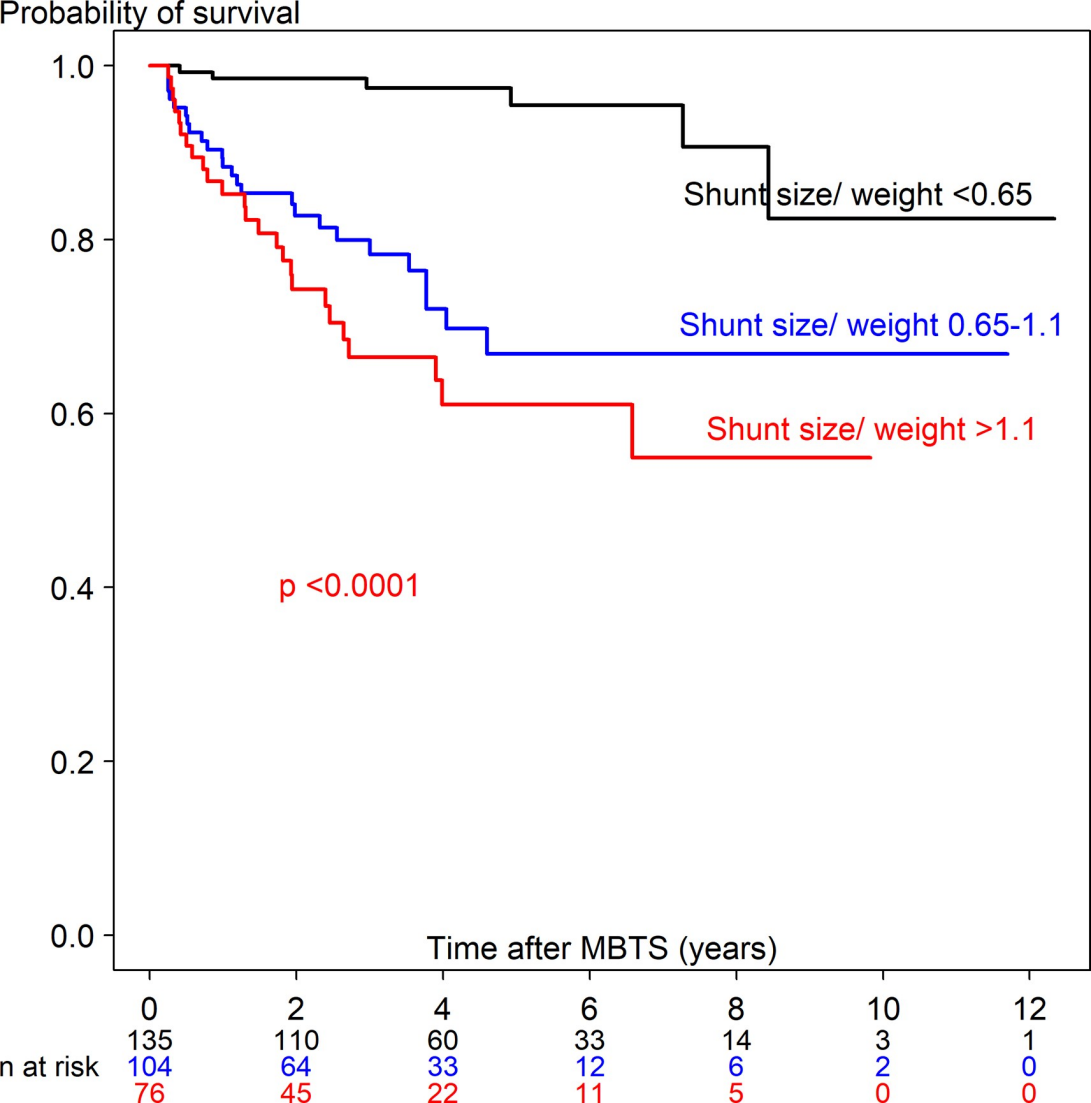
Kaplan-Meier curve of time-to-death >90 days after receiving a modified Blalock-Taussig shunt among three shunt size/weight ratios.

S1 Table shows the univariate Cox regression analysis predicting time-to-death ≤90 days (63 deaths out of 380) and >90 days (56 deaths out of 315). Potential predictors from the univariate analysis included 28 covariates for time-to-death ≤90 days and 21 covariates for time-to-death >90 days. Figs 3 and 4 show the Kaplan-Meier curve of shunt size/weight ratio 0.65–1.1 and >1.1 compared to <0.65 for time-to-death ≤90 days and >90 days. The multivariate Cox regression model with adjustment for time-dependent covariates is shown in Table 3. After stepwise variable selection, 22 variables for time-to-death ≤90 days and 10 variables for time-to-death >90 days remained in the final model. The predictors of time-to-death ≤90 days were related with comorbidity (weight <3 kg, prematurity, preoperative ventilator support), intraoperative cardiac arrest and hypoxemia with bradycardia and postoperative complications (shunt thrombosis, bleeding, renal failure and sepsis). Protective factors of time-to-death ≤90 days were increased difference in postoperative and preoperative oxygen saturation and shunt size/weight ratio >1.1 vs <0.65.

**Table 3:** Predictors of time to death ≤90 days and >90 days by time dependent multivariate Cox proportional hazard model (n = 695).

Variable	Death ≤90 days (n = 380, deaths = 63)		Death >90 days (n = 314, deaths = 56)	
	Adjusted HR (95% CI)	p-value*	Adjusted HR (95% CI)	p-value*
**Preoperative period**				
Age ≤1 month (ref = >12)	0.96 (0.21, 4.34)	0. 960	0.38 (0.11, 1.33)	0.131
Age >1–12 month (ref = >12)	3.09 (0.92, 10.4)	0.068	0.31 (0.09, 1.05)	0.060
Body weight <3 kg	7.60 (2.83, 20.4)	<0.0001	2.11 (0.84, 5.33)	0.113
History of prematurity	2.05 (1.02, 4.12)	0.044	-	
Dextrocardia	1.83 (0.80, 4.16)	0.150	1.89 (0.81, 4.42)	0.142
Ventilator support	2.67 (1.28, 5.60)	0.009	-	
Complex heart (ref = TOF)	2.92 (1.00, 8.58)	0.051	1.25 (0.25, 6.33)	0.587
Single ventricle (ref = TOF)	1.53 (0.68, 3. 41)	0.302	3.09 (1.26, 7.56)	0.014
PA-VSD (ref = TOF)	0.74 (0.25, 2.17)	0.583	3.17 (1.23, 7.72)	0.011
Inotrope use 1 agent (ref = no)	0.66 (0.27, 1.63)	0.367	1.68 (0.82, 3.47)	0.159
Inotrope use >1 agent (ref = no)	2.43 (0.69, 8.52)	0.166	-	
**Intraoperative period**				
Shunt size/weight ratio 0.65–1.1 (ref = <0.65)	0.84 (0.27, 2.62)	0.764	10.3 (2.97, 35.7)	0.0002
Shunt size/weight ratio >1.1 (ref = <0.65)	0.12 (0.02, 0.66)	0.014	6.81 (1.42, 32.6)	0.016
Cardiac failure	0.15 (0.02, 1.26)	0.081	-	
Cardiac arrest	121.5 (15.5, 950.5)	<0.0001	-	
Hypoxemia with bradycardia (ref = no)	4.1 (1.56, 10.36)	0.004	-	
Hypoxemia without bradycardia (ref = no)	0.71 (0.34, 1.49)	0.364	-	
Duration of surgery (minutes)	1. 013 (1.004, 1.023)	0.006	-	
**Postoperative period**				
PostSpO_2_ –preSpO_2_	0.979 (0.960, 0.999)	0.039	0.972 (0.943, 1.004)	0.083
Duration of mechanical ventilator (hours)	-		1.002 (1.001, 1.004)	0.008
Length of hospital stay (days)	-		0.979 (0.957, 1.001)	0.060
Shunt thrombosis	4.96 (2.37, 10.39)	<0.0001	-	
Bleeding	4.49 (2.13, 9.42)	<0.0001	-	
Sepsis	2.73 (1.27., 5.85)	0.010	-	
Renal failure	4.07 (1.52, 10.91)	0.005	-	
Perigraft seroma/chylothorax	0.12 (0.01, 0.96)	0.046		
Readmission within 30 days (ref = no)	2.96 (0.49, 17.88)	0.237	-	
Admission > 30 days (ref = no)	0.40 (0.20, 0.81)	0.010	-	
Admission > 90 days (ref = no)	-		17. 25 (2.11, 140.8)	0.008

*Wald's test. TOF, tetralogy of fallot; HR, hazard ratio; CI, confidence interval; PA-VSD, pulmonary atresia with ventricular septal defect; PostSpO_2_, postoperative oxygen saturation; preSpO_2_, preoperative oxygen saturation; ICU, intensive care unit.

The predictors of time-to-death >90 days (56 children) were related with particular cardiac defect (PA-VSD and single ventricle [versus TOF], larger shunt size/weight ratio (0.65–1.1 and >1.1 [versus <0.65], longer hours of ventilator support and admission >90 days. Three neonates (4 days old, 6-days old and 23 days-old) had a length of stay more than 90 days and died after being discharged—2 died from sepsis (shunt size/weight ratio 1.1 and 1.2) and the other from cardiac failure (shunt size/weight ratio 1.6).

Table 4 lists the causes of death. Most died from cardiac failure (23%), severe hypoxic spell (20%), and sepsis (18%).

**Table 4:** Cause of death ≤90 days and >90 days after receiving modified Blalock-Taussig shunt (n = 119).

Cause of death	≤90 days (n = 63)	> 90 days (n = 56)
Congestive heart failure	29 (46.0%)	13 (23.2%)
Shunt thrombosis	13 (20.6%)	3 (5.4%)
Severe sepsis	10 (15.9%)	10 (17.9%)
Severe hypoxic spell	7 (11.1%)	11 (19.6%)
Hypoxic arrest from other causes	7 (11.1%)	2 (3.6%)
Bleeding	7 (11.1%)	1 (1.8%)
Multiorgan failure	1 (1.6%)	1 (1.8%)
Cerebral hemorrhage/infarction	0	2 (3.6%)
Hyperkalemia	1 (1.6%)	0
Severe pulmonary artery hypertension	1 (1.6%)	0
Drug overdose (heparin, digitalis)	0	1 (1.8%)
Unknown	0	14 (25.0%)

### Subgroup analysis of age-specific time-to-death after receiving MBTS

S5–S7 Figs show the Kaplan-Meier curves of shunt size/weight ratio and time-to-death in neonates (≤1 month), infants (>1–12 months) and toddlers (>12 months), respectively. The optimal cut-points for shunt size/weight ratio and time-to-death in neonates, infants and toddlers were 1.1, 0.7 and 0.58, respectively. Therefore, shunt size/weight ratio ≥1.0 (versus <1.0) for neonates and ≥0.65 (versus <0.65) for infants and toddlers were created and included in the univariate Cox regression model with the results shown in S2 Table. Potential predictors with a p-value ≤0.1 and included in the multivariate Cox regression analysis were 16 covariates for overall time-to-death in neonates, 12 covariates for overall time-to-death in infants and 14 covariates in toddlers (age >12 months). The multivariate Cox proportional hazard model for each subgroup with adjustment for these covariates is shown in S3 Table. The common risk factors for time-to-death in all subgroups was postoperative sepsis. The common risk factors for time-to-death in neonates and toddlers were postoperative shunt thrombosis and larger shunt size/weight ratio (≥1.0 for neonates and ≥0.65 for toddlers).

## Discussion

The overall mortality rate (31%), including in-hospital deaths (15.5%) and interim deaths (waiting for the next stage, 15.8%), after MBTS at our hospital was relatively high compared to other studies [3, 13]. Alsoufi et al. [13] reported an in-hospital mortality rate of 15% for single ventricle and 23% for PAIVS in neonates. Chittithavorn et al. [8] reported an in-hospital mortality rate of 22% for PAIVS in our hospital after MBTS in low weight neonates. In this study we included children aged up to 3 years old and the in-hospital mortality rate was reasonable considering that palliative shunt in cyanotic heart children in our setting which most causes of death were related to complications after receiving MBTS (Table 4). Alsoufi et al. [3] reported an interim death rate of 10% in infants followed up for 2 years after MBTS in single ventricle and biventricular cardiac anomalies. In our study, we included some children whose palliative shunt (MBTS) was given as definitive treatment (<20%) and followed children for 12 years after MBTS, and even though after 1.5 years the death rate was quite low (Fig 2), children who did not fit/or were waiting for the definite repair, were still followed and all interim deaths occurred at around 8 years after MBTS.

The proportion of children receiving Glenn/biventricular repair increased around 5 months and peaked at 6.5 years (42.3%). The optimal age for the Glenn procedure is around 6–12 months (5–10 kg) in our institute which is consistent with other studies [14, 15] whereas performing biventricular repair depends on the cardiac diagnosis [16–18]. However, some children had a very long delay before definite repair in our institute which could arise from many reasons. First, since Songklanagarind Hospital is the only referral center for complex heart surgery in southern Thailand, most cases, after receiving a palliative shunt, are referred back to their hometown. The referral system between our hospital and hometown hospitals is underdeveloped. Secondly, most of the cases came from low socioeconomic families, which tend to have a high attrition rate. Other problems include a limited number of surgeons, competing operating room queues with adult cases and an unavailability/insufficiency of PICU beds. However, a total of 139 children (36.6%) received Glenn/biventricular repair and 122 (32%) still survived without definite repair within 12 years of follow up.

Since the highest period of time-to-death after MBTS was 90 days (Fig 2), we split the data into time-to-death *≤*90 days (n = 380) and >90 days (n = 315) after MBTS using time-dependent survival analysis.

### Risk factors of time-to-death ≤90 days

The predictors for time-to-death ≤90 days are shown in Table 3. Low weight at the time of MBTS and history of prematurity were more important risk factors than age group to predict time-to-death ≤90 days after MBTS. We found that children weighing <3 kg and premature infants had a higher hazard rate of time-to-death ≤90 days (7.6-fold and 2.1-fold, respectively) which is consistent with other studies [4, 5, 19]. Higher ASA classification was not a significant predictor for time-to-death in our study even though it increased postoperative mortality in patients with congenital heart disease in other studies [20, 21]. This may be because specific patient coexisting diseases to define ASA classification were significant risk factors (2.7-fold for preoperative ventilator support and 11-fold for use of at least 2 inotropes). Postoperative medical complication associated with time-to-death ≤90 days in our study (2.7-fold for sepsis and 4-fold in renal failure) were supported by other studies in neonates [7, 22] whereby postoperative sepsis increasing the risk of overall time-to-death was confirmed by our subgroup analysis (S3 Table).

Postoperative surgical complications increased hazard rate of time-to-death ≤90 days in our study (4.5-fold for bleeding and 5.0-fold for shunt thrombosis) which was supported by cause of death in Table 4 and other studies [7, 8, 23]. Moreover, we found that shunt size/weight ratio >1.1 (compared to <0.65) decreased the hazard rate of time-to-death ≤90 days (0.12-fold, p = 0.014). This finding implies that lower shunt size/weight ratio (<0.65) might be associated with shunt problems, for example shunt occlusion or shunt thrombosis leading to death within 90 days after MBTS.

### Risk factors of time-to-death >90 days

The predictors for time-to-death >90 days were shown in Table 3. According to Table 3 and S3 Table, shunt size/weight ratio >1.1 increased the hazard rate of time-to-death >90 days (7-fold) and in the neonate subgroup (13-fold), whereas shunt size/weight ratio 0.65–1.1 increased the hazard rate of time-to-death >90 days (10-fold) and in toddlers (8-fold). Kucuk et al. [23] reported shunt size/weight ratio of 1.25 related to overcirculation in infants (≤20 months) after 1 day to 48 months follow up from MBTS with no deaths. Our study followed children 1–12 years after MBTS and found the cut-point, which was related to time-to-death in the neonate (1.0) and toddler (0.65) subgroups. The higher shunt size/weight ratio may be associated with shunt overflow in the long run leading to cardiac failure and death >90 days. For toddlers, shunt size/weight ratio ≥0.65 associated with time-to-death might be related to shunt problems, for example shunt thrombosis or shunt overflow. Even though cardiac failure seemed to be the main cause of death after 90 days, most cardiac failure cases were due to poor cardiac function itself related with a particular cardiac defect rather than from over-circulation. We found that longer mechanical ventilation time increased the hazard rate (1.002 times per hour) which was related to postoperative shunt thrombosis, VAP and sepsis.

In studies from Libya and USA, diagnosis other than TOF was the principal risk factor for early mortality [24, 25], which was consistent with our study. We found that children with a single ventricle (majority was PAIVS), compared to TOF, had an increased hazard of time-to-death >90 days (3.1-fold) and overall time-to-death in infants (3.3-fold). Studies have shown that children with PAIVS or a single ventricle had the poorest outcome in terms of shunt failure and mortality [3, 5]. PAIVS children have a higher incidence of ventricular-coronary artery sinusoids or fistula which could result in a higher risk of myocardial ischemia, cardiac failure/cardiac arrest or shunt problems after MBTS [13, 26, 27]. PA-VSD (compared to TOF) also increased the hazard rate of time-to-death >90 days (3.2-fold) in our study. Lertsakulpiriya et al. [28] reported that the survival of children with PA-VSD was as high as 95% at 1 year, but decreased to 83.7% at 5 years. PA-VSD was found to be associated with major aortopulmonary collateral arteries [29] in which competitive flow of MBTS might occur leading to shunt problems, hypoxic spell, ventricular failure and eventually death.

### Protective factors of time-to-death

Increase in post shunt SpO_2_ levels from baseline reduced the hazard rate of time-to-death *≤*90 days (p = 0.039) and overall time-to-death in all subgroups (p = 0.006 for neonates, p = 0.009 for infants and p = 0.007 for toddlers). Previous studies found that rising postoperative PaO_2_ levels played a crucial role in postoperative weaning and recovery [4, 30].

### Implications for clinical practice

Since preoperative factors and perioperative complications were the predictors for time-to-death ≤90 days, minimizing intraoperative (hypoxemia and bradycardia) and postoperative complications (thrombosis and bleeding) might be possible if effectively incorporated by cardiovascular thoracic surgeons, anesthesiologists and pediatric cardiologists in the operating room and at hand over in the PICU. Appropriate shunt size (shunt size/weight ratio <1.0 for neonates) and evaluation of shunt function is necessary to prevent shunt thrombosis.

Management of anticoagulants should be weighed against the risk of shunt thrombosis and risk of bleeding. To minimize the risk of death >90 days, neonates who have a shunt size/weight ratio ≥1.0 and >0.65 in older aged children should be followed up within 3 months to evaluate shunt overflow, especially in particular cardiac defects (single ventricle, PA-VSD) to consider for Glenn/total repair. In our practice, children were scheduled to meet at the surgical department 2 weeks after discharge from hospital and then were scheduled to meet a pediatric cardiologist every 3–6 months depending on the severity of the particular cardiac defect and shunt function. If shunt thrombosis/occlusion is diagnosed, cardiac catheterization with balloon angioplasty is readily available. Moreover, our team hold regular monthly cardiovascular, thoracic and pediatric conferences to discuss urgent cases to determine which period should be the optimal time for definite repair after an MBTS.

### Strengths and limitations

The strengths of this study are as follows. First, we used a competing risk survival and time-dependent variables survival analysis to examine changing risks by time (≤90 and >90 days) affecting death after receiving MBTS. Second, we used multivariate analysis to adjust for the effect of confounders. Third, we performed subgroup analyses for children of different age groups to explore risk factors that might be different. However, some limitations in the study need to be acknowledged. First, our historical cohort design may have resulted in information bias from under-reporting. Second, each patient received different follow up times (1–12 years) which may have affected time-to death or causes of death >90 days in some patients due to unknown medical or non-medical causes. Finally, recruitment of cases from a single center limits the generalizability of the study findings.

## Conclusions

Preoperative conditions and postoperative complications were associated with time-to-death ≤90 days, whereas specific cardiac conditions, larger shunt size and longer hours of mechanical ventilation were associated with time-to-death >90 days. Incorporation of a multidisciplinary team to prevent perioperative complications, especially in high-risk children and high risk cardiac diagnoses, might improve patient survival after receiving MBTS. Shunt size/weight ratio >1.0 for neonates and >0.65 for children aged >1 year should be reevaluated within 90 days to reduce the risk of shunt over flow or clinical cardiac failure.

## Supporting information

S1 Fig Kaplan-Meier curve of the time-to-death ≤90 days after receiving a modified Blalock-Taussig shunt (A). Nelson Aalen plot for cumulative hazard of the event of death ≤90 days after receiving a modified Blalock-Taussig shunt (B).
(TIF)

S2 Fig Kaplan-Meier curve of the time-to-death >90 days after receiving a modified Blalock-Taussig shunt (A). Nelson Aalen plot cumulative hazard of the event of death >90 days after receiving a modified Blalock-Taussig shunt (B).
(TIF)

S3 Fig Kaplan-Meier survival curve of shunt size/weight ratio among children who died ≤90 days. (TIFF)

S4 Fig Kaplan-Meier survival curve of shunt size/weight ratio among children who died >90 days. (TIFF)

S5 Fig The Kaplan-Meier survival curve of shunt size/weight ratio in subgroup neonates. (TIFF)

S6 Fig The Kaplan-Meier survival curve of shunt size/weight ratio in subgroup infants. (TIFF)

S7 Fig The Kaplan-Meier survival curve of shunt size/weight ratio in subgroup toddler. (TIFF)

S1 Table Univariate Cox regression analysis for time to death ≤90 days and >90 days compared to censor (n = 695). * P value by Wald test, ASA, American Society of Anesthesiologists; TOF, tetralogy of fallot; HR, hazard ratio; CI, confidence interval; MBTS, modified Blalock-Taussig shunt; PostSpO_2_, postoperative oxygen saturation; preSpO_2_, preoperative oxygen saturation; ICU, intensive care unit; PA-VSD, pulmonary atresia with ventricular septal defect.
(DOCX)

S2 Table Subgroup univariate Cox analysis for time to death in children aged ≤1 month, aged between 1 month and 1 year, and aged 1–3 years. *by Wald test, ASA, American Society of Anesthesiologists; TOF, tetralogy of fallot; HR, hazard ratio; CI, confidence interval; MBTS, modified Blalock-Taussig shunt; PostSpO_2_, postoperative oxygen saturation; preSpO_2_, preoperative oxygen saturation; ICU, intensive care unit; PA-VSD, pulmonary atresia with ventricular septal defect.
(DOCX)

S3 Table Subgroup analysis predictors for time to death in children aged ≤1 month, and aged >1 month by time dependent multivariate Cox proportional hazard model (n = 380). * p values were calculated using the Cox-proportional hazard model (Wald test), HR, hazard ratio; CI, confidence interval; MBTS, modified Blalock-Taussig shunt; PostSpO_2_, postoperative oxygen saturation; PreSpO_2_, preoperative oxygen saturation; TOF, tetralogy of fallot; PA-VSD, pulmonary atresia with ventricular septal defect.
(DOCX)

S1 File Maliwan380.Rds. (CSV)

S2 File Maliwanless90.Rds. (CSV)

S3 File Maliwanmore90.Rds. (CSV)
