# Supplementary figures and images for: Predictors of death after receiving a modified Blalock-Taussig shunt in cyanotic heart children: A competing risk analysis
Source: PLoS One. 2021 Jan 22;16(1):e0245754. doi: 10.1371/journal.pone.0245754 (PMC7822344; doi:10.1371/journal.pone.0245754)

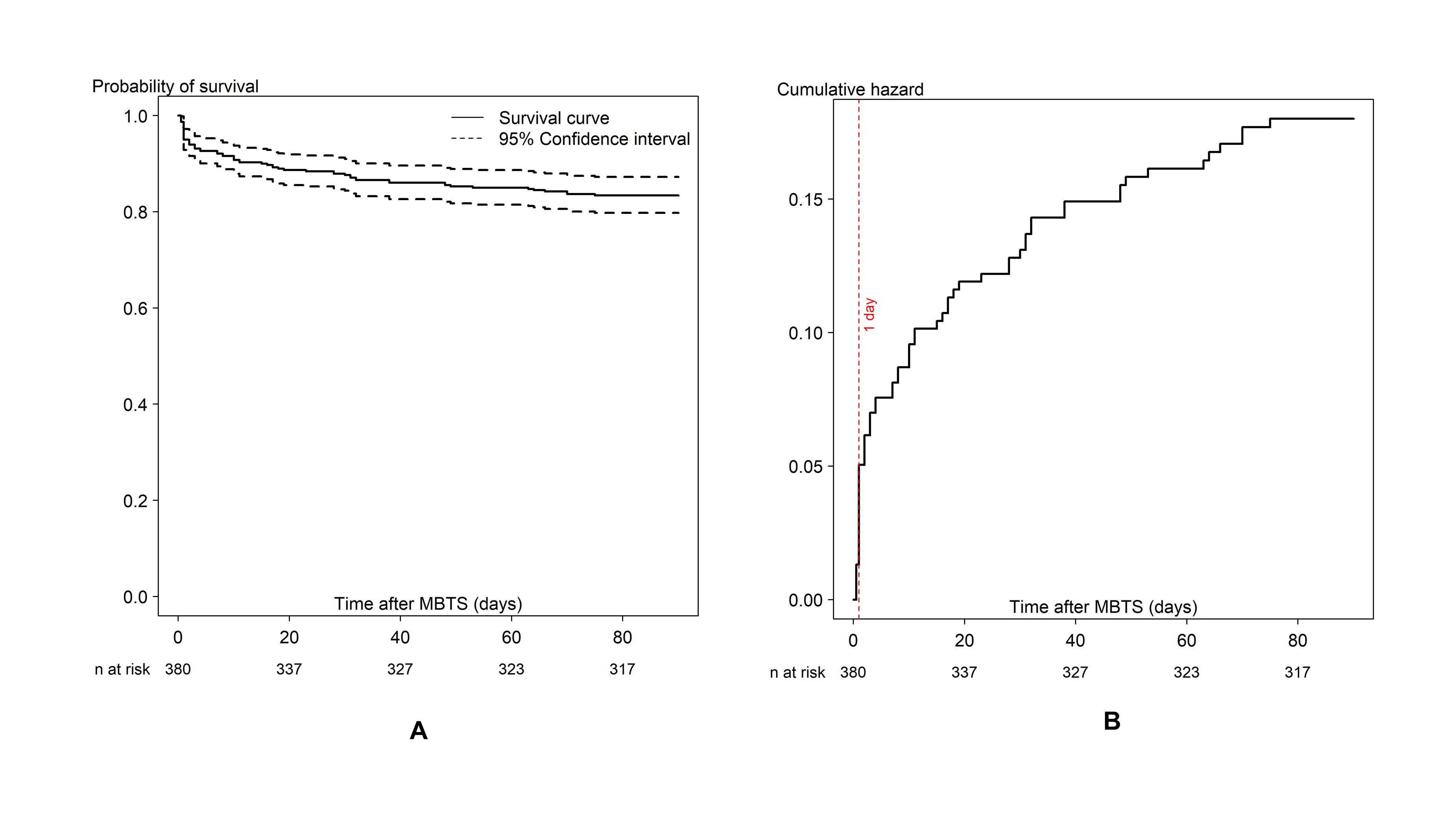

Supplement: S1 Fig — Kaplan-Meier curve of the time-to-death ≤90 days after receiving a modified Blalock-Taussig shunt (A). Nelson Aalen plot for cumulative hazard of the event of death ≤90 days after receiving a modified Blalock-Taussig shunt (B). (TIF) [file pone.0245754.s001.tif]

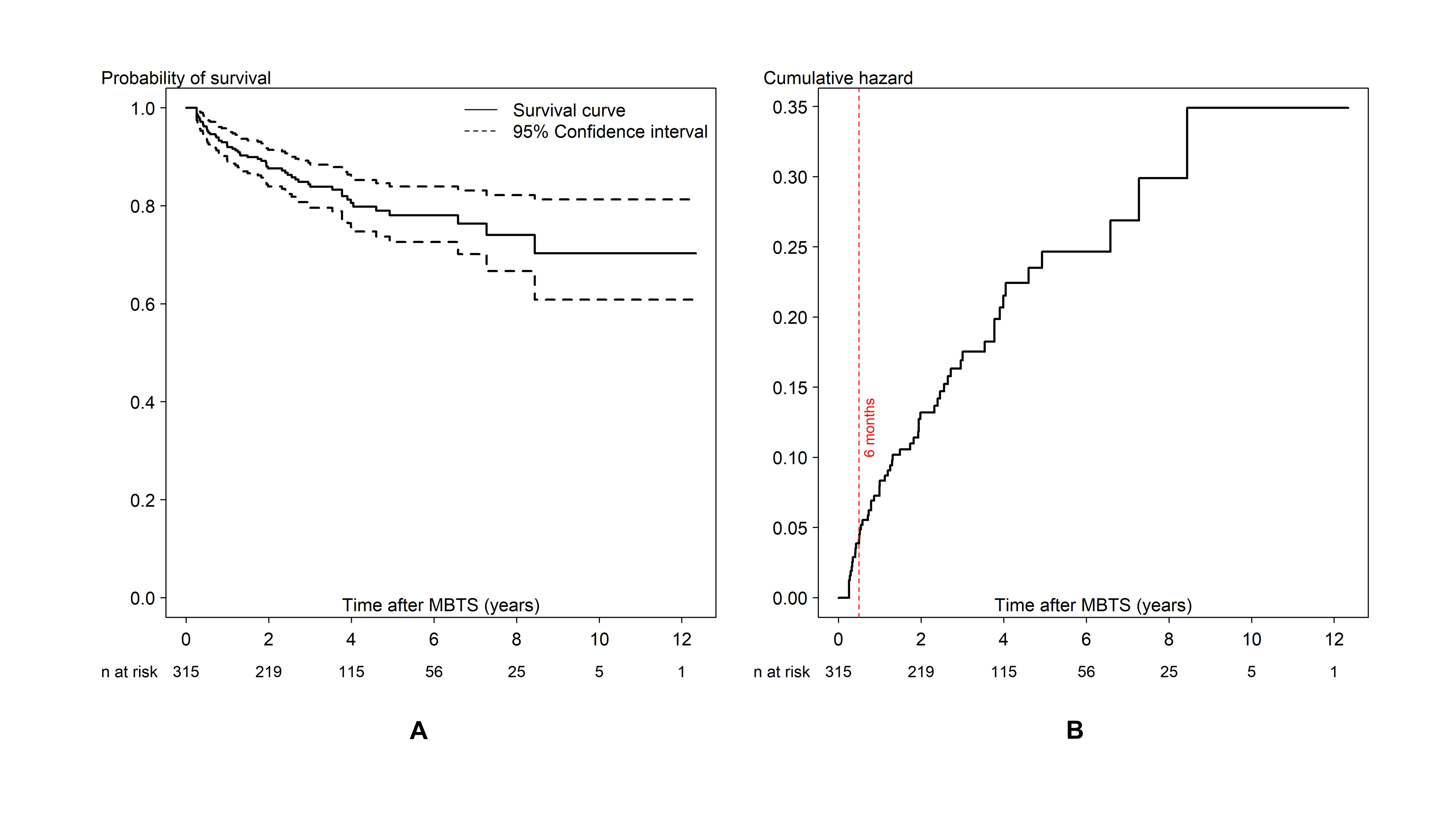

Supplement: S2 Fig — Kaplan-Meier curve of the time-to-death >90 days after receiving a modified Blalock-Taussig shunt (A). Nelson Aalen plot cumulative hazard of the event of death >90 days after receiving a modified Blalock-Taussig shunt (B). (TIF) [file pone.0245754.s002.tif]

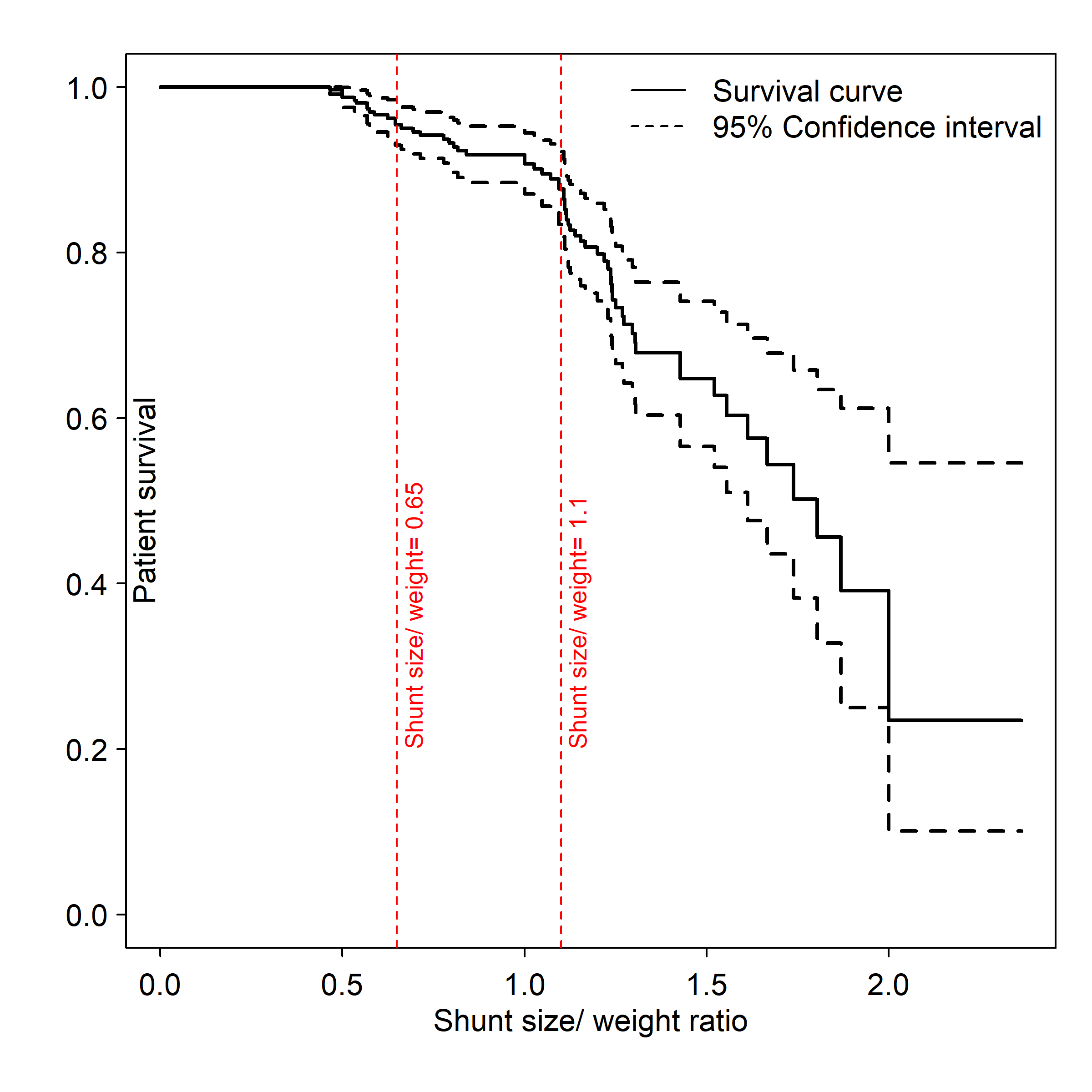

Supplement: S3 Fig — (TIFF) [file pone.0245754.s003.tiff]

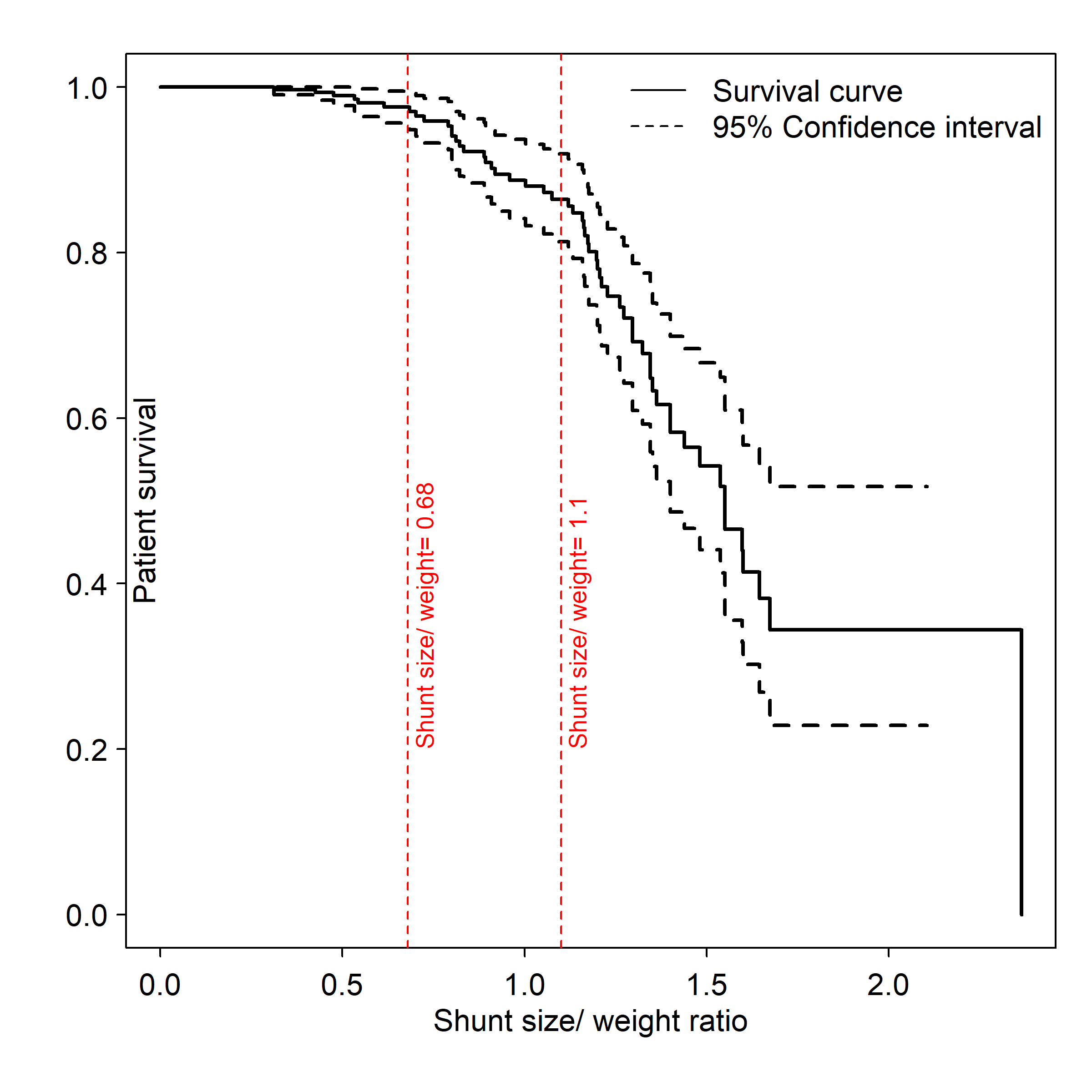

Supplement: S4 Fig — (TIFF) [file pone.0245754.s004.tiff]

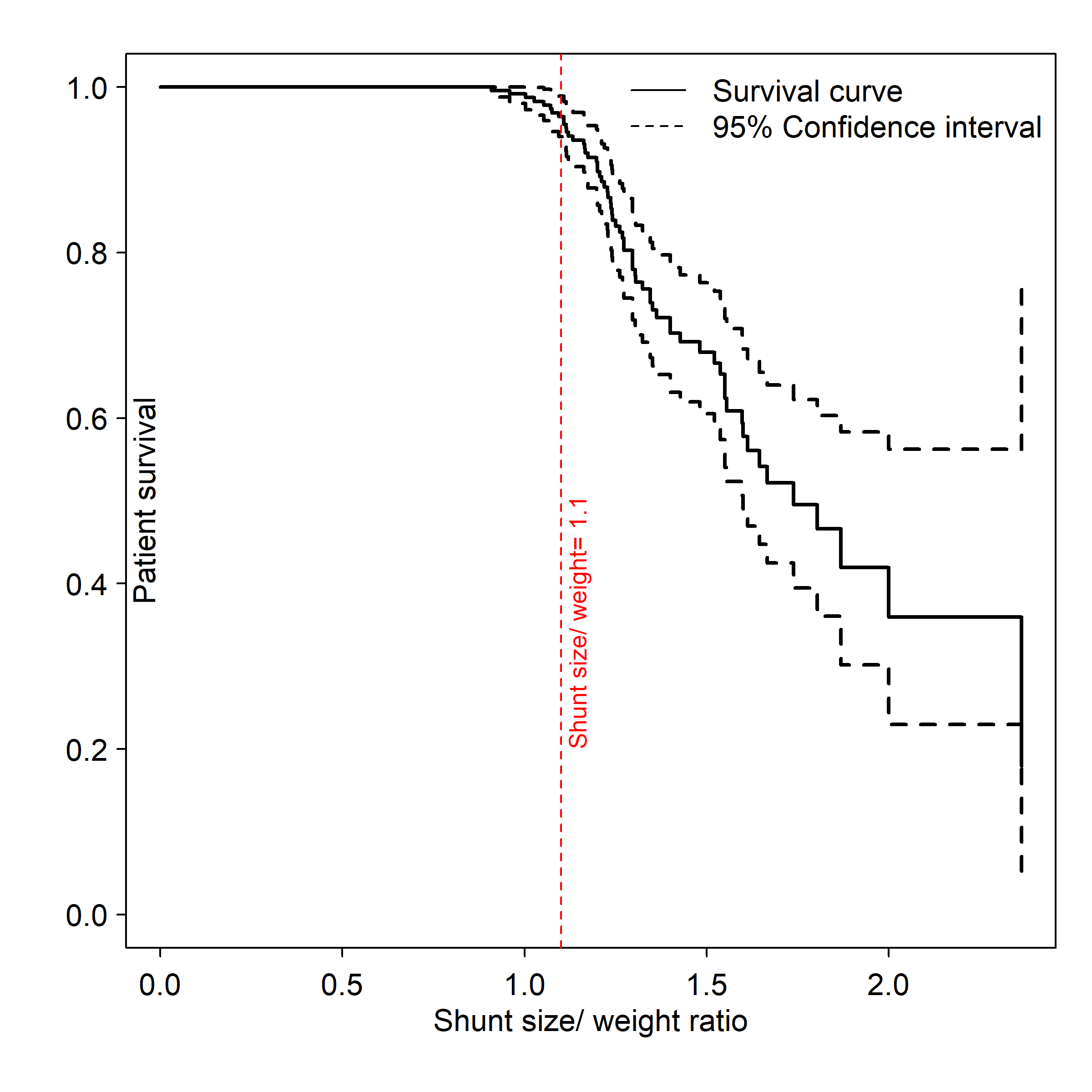

Supplement: S5 Fig — (TIFF) [file pone.0245754.s005.tiff]

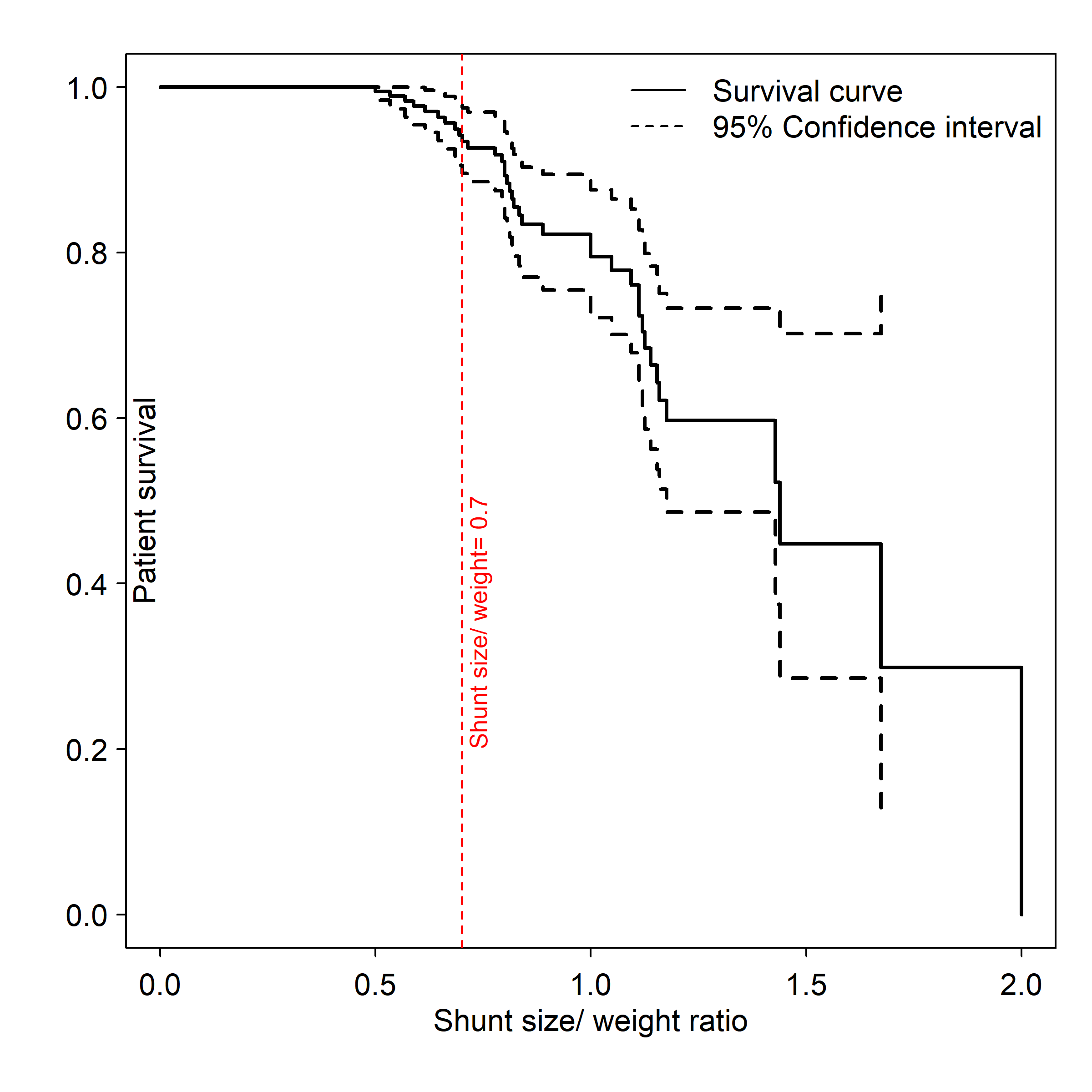

Supplement: S6 Fig — (TIFF) [file pone.0245754.s006.tiff]

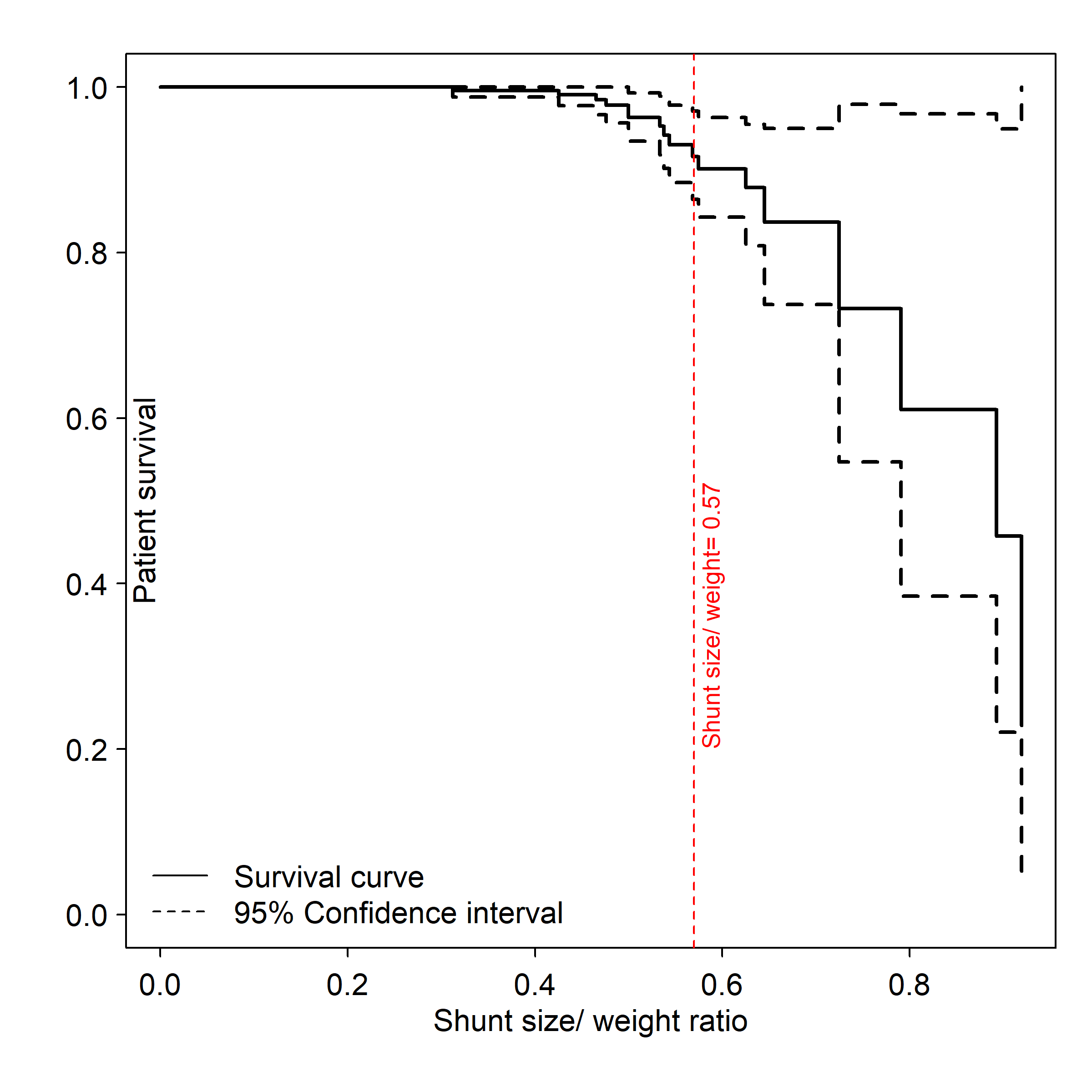

Supplement: S7 Fig — (TIFF) [file pone.0245754.s007.tiff]
