# Supplementary material for: Predictors of death after receiving a modified Blalock-Taussig shunt in cyanotic heart children: A competing risk analysis
Source: PLoS One. 2021 Jan 22;16(1):e0245754. doi: 10.1371/journal.pone.0245754 (PMC7822344; doi:10.1371/journal.pone.0245754)
Supplement: S1 Table — * P value by Wald test, ASA, American Society of Anesthesiologists; TOF, tetralogy of fallot; HR, hazard ratio; CI, confidence interval; MBTS, modified Blalock-Taussig shunt; PostSpO2, postoperative oxygen saturation; preSpO2, preoperative oxygen saturation; ICU, intensive care unit; PA-VSD, pulmonary atresia with ventricular septal defect. (DOCX) [file pone.0245754.s008.docx]

**S1 Table. Univariate Cox regression analysis for time to death ≤90 days and >90 days compared to censor (N=695)**

| **Variables** | **Death ≤90 days (n=380)**  **Adjusted HR**  **(95% CI)** | **p value*** | **Death >90 days (n=315)**  **Adjusted HR**  **(95% CI)** | **p value*** |
| --- | --- | --- | --- | --- |
| **Preoperative** **period** |  |  |  |  |
| Age ≤1 months (ref= >12) | 3.67 (1.68, 8.02) | 0.001 | 5.55 (2.65, 11.62) | <0.0001 |
| Age >1-12 months (ref= >12) | 4.00 (1.80, 8.87) | 0.0006 | 2.38 (1.02, 5.56) | 0.046 |
| Body weight <3 kg | 2.16 (1.31, 3.59) | 0.003 | 3.25 (1.92, 5.50) | <0.0001 |
| History of prematurity | 2.02 (1.14, 3.56) | 0.015 | 0.89 (0.40, 1.97) | 0.777 |
| Heterotaxy syndrome | 1.90 (0.91, 4.00) | 0.089 | 1.68 (0.67, 4.23) | 0.267 |
| Others syndrome | 0.60 (0.19, 1.90) | 0.381 | 0.32 (0.08, 1.32) | 0.116 |
| Dextrocardia | 2.18 (1.08, 4.41) | 0.031 | 2.14 (0.97, 4.74) | 0.060 |
| Chronic lung disease | 1.30 (0.66, 2.55) | 0.451 | 1.14 (0.54, 2.41) | 0.731 |
| Sepsis | 2.37 (1.02, 5.50) | 0.044 | 0.38 (0.05, 2.77) | 0.342 |
| Hypoxic spell | 2.82 (1.02, 7.76) | 0.045 | 2.43 (0.59, 9.98) | 0.219 |
| Congestive heart failure | 2.62 (1.05, 6.53) | 0.039 | 1.55 (0.38, 6.37) | 0.544 |
| Ventilator support | 3.82 (2.33, 6.27) | <0.0001 | 1.88 (1.06, 3.33) | 0.030 |
| Complex heart (ref = TOF) | 4.23 (1.83, 9.77) | 0.0007 | 2.07 (0.44, 9.78) | 0.358 |
| Single ventricle (ref = TOF) | 2.91 (1.47, 5.75) | 0.002 | 5.34 (2.42, 11.77) | <0.0001 |
| PA-VSD (ref = TOF) | 1.15 (0.46, 2.87) | 0.76 | 3.59 (1.56, 8.26) | 0.003 |
| Prostaglandin E1 use | 2.01 (1.23, 3.30) | 0.006 | 4.45 (2.59, 7.66) | <0.0001 |
| Times of having MBTS | 0.53 (0.22, 1.25) | 0.147 | 0.32 (0.10, 1.01) | 0.053 |
| Inotrope use 1 agent (ref=No) | 2.01 (1.11, 3.62) | 0.021 | 1.85 (0.98, 3.51) | 0.058 |
| Inotrope use > 1 agent (ref=No) | 7.85 (3.33, 18.49) | <0.0001 | - |  |
| ASA classification 4 (ref=2 and 3) | 1.56 (1.20, 2.02) | 0.0008 | 1.25 (0.92, 1.71) | 0.159 |
| Emergency case (ref=elective) | 2.64 (1.38, 5.06) | 0.003 | 2.44 (1.29, 4.62) | 0.006 |
| **Intraoperative period** |  |  |  |  |
| Inotrope use 1 agent (ref=No) | 1.37 (0.71, 2.62) | 0.346 | 0.96 (0.53, 1.77) | 0.906 |
| Inotrope use > 1 agent (ref=No) | 1.85 (0.85, 3.99) | 0.120 | 1.02 (0.45, 2.32) | 0.960 |
| Hemodilution | 0.47 (0.12, 1.92) | 0.294 | 0.47 (0.12, 1.94) | 0.298 |
| Shunt size/ weight ratio 0.65-1.1 (ref=<0.65) | 2.33 (1.20, 4.53) | 0.013 | 6.71 (2.75, 16.38) | <0.0001 |
| Shunt size/ weight ratio >1.1 (ref=<0.65) | 2.97 (1.51, 5.83) | 0.002 | 9.49 (3.89, 23.17) | <0.0001 |
| Hypoxemia with bradycardia (ref=No) | 3.67 (1.70, 7.96) | 0.001 | 1.86 (0.66, 5.22) | 0.239 |
| Hypoxemia without bradycardia (ref=No) | 2.12 (1.23, 3.63) | 0.006 | 1.59 (0.87, 2.91) | 0.129 |
| Cardiac failure | 45.67 (17.14, 121.7) | <0.0001 | - |  |
| Cardiac arrest | 24.50 (11.00, 54.58) | <0.0001 | - |  |
| Blood loss (ml) | 1.000 (0.994, 1.008) | 0.828 | 0.980 (0.960, 1.003) | 0.093 |
| Duration of surgery (minutes) | 1.007 (1.000, 1.015) | 0.069 | 0.995 (0.986, 1.004) | 0.296 |
| **Postoperative period** |  |  |  |  |
| PostSpO_2_ – preSpO_2_ | 0.945 (0.930, 0.960) | <0.0001 | 0.967 (0.941, 0.994) | 0.015 |
| Duration of mechanical ventilator (days) | 1.000 (0.999, 1.002) | 0.538 | 1.002 (1.001, 1.003) | <0.0001 |
| Length of ICU stay (days) | 0.998 (0.983, 1.014) | 0.844 | 1.016 (1.008, 1.024) | <0.0001 |
| Length of hospital stay (days) | 0.997 (0.984, 1.010) | 0.602 | 1.013 (1.004, 1.023) | 0.004 |
| Shunt thrombosis | 5.37 (3.19, 9.04) | <0.0001 | 1.66 (0.71, 3.87) | 0.243 |
| Bleeding | 7.27 (4.26, 12.42) | <0.0001 | 1.44 (0.45, 4.62) | 0.539 |
| Pneumothorax | 1.54 (0.76, 3.13) | 0.228 | 2.76 (1.39, 5.50) | 0.004 |
| Pneumonia | 1.46 (0.86, 2.46) | 0.157 | 1.54 (0.87, 2.73) | 0.137 |
| Renal failure | 10.15 (4.94, 20.84) | <0.0001 | - |  |
| Sepsis | 5.18 (3.13, 8.57) | <0.0001 | 2.69 (1.36, 5.34) | 0.005 |
| Chylothorax/ perigraft seroma | 0.19 (0.03, 1.36) | 0.098 | 0.58 (0.18, 1.85) | 0.357 |
| Shunt revision | 2.71 (1.44, 5.08) | 0.002 | 2.16 (1.02, 4.57) | 0.044 |
| Numbers of reoperative thoracotomy during admission | 1.70 (1.29, 2.24) | 0.0002 | 1.43 (0.99, 2.05) | 0.057 |
| Readmission within 30 day (ref=No) | 2.74 (0.66, 11.30) | 0.164 | 2.55 (0.35, 18.63) | 0.357 |
| Admission > 30 days (ref=No) | 1.68 (0.96, 2.94) | 0.070 | 2.33 (1.28, 4.22) | 0.005 |
| Admission > 90 days (ref=No) | - |  | 5.02 (1.55, 16.27) | 0.007 |
| Total times of MBTS and other surgeries related to MBTS in all admission | 0.95 (0.68, 1.32) | 0.750 | 0.77 (0.52, 1.14) | 0.193 |

* P value by Wald test, ASA, American Society of Anesthesiologists; TOF, tetralogy of fallot; HR, hazard ratio; CI, confidence interval; MBTS, modified Blalock-Taussig shunt; PostSpO_2_, postoperative oxygen saturation; preSpO_2_,preoperative oxygen saturation; ICU, intensive care unit; PA-VSD, pulmonary atresia with ventricular septal defect
