# Supplementary material for: Predictors of death after receiving a modified Blalock-Taussig shunt in cyanotic heart children: A competing risk analysis
Source: PLoS One. 2021 Jan 22;16(1):e0245754. doi: 10.1371/journal.pone.0245754 (PMC7822344; doi:10.1371/journal.pone.0245754)
Supplement: S2 Table — *by Wald test, ASA, American Society of Anesthesiologists; TOF, tetralogy of fallot; HR, hazard ratio; CI, confidence interval; MBTS, modified Blalock-Taussig shunt; PostSpO2, postoperative oxygen saturation; preSpO2, preoperative oxygen saturation; ICU, intensive care unit; PA-VSD, pulmonary atresia with ventricular septal defect. (DOCX) [file pone.0245754.s009.docx]

**S2 Table. Subgroup univariate Cox analysis for time to death in children aged ≤1 month, aged between 1 month and 1 year, and aged 1-3 year**

| **Variables** | **Aged ≤1 month (n=142, death 63)**  **Adjusted HR (95% CI)** | **p value*** | **Aged between 1 month and 1 year (n=109, death 38)**  **Adjusted HR (95% CI)** | **p value*** | **Aged 1-3 year (n=129, death 17)**  **Adjusted HR (95% CI)** | **p value*** |
| --- | --- | --- | --- | --- | --- | --- |
| **Preoperative** **period** |  |  |  |  |  |  |
| Body weight <3 kg | 1.74 (1.03, 2.96) | 0.040 | 1.56 (0.65, 3.75) | 0.32 | - |  |
| History of prematurity | 1.09 (0.57, 2.09) | 0.796 | 1.86 (0.90, 3.84) | 0.092 | 0.99 (0.23, 4.34) | 0.987 |
| Heterotaxy syndrome | 1.76 (0.80, 3.90) | 0.161 | 1.97 (0.82, 4.72) | 0.130 | - |  |
| Others syndrome | 0.41 (0.10, 1.6) | 0.218 | 0.66 (0.16, 2.76) | 0.572 | 0.50 (0.07, 3.79) | 0.504 |
| Dextrocardia | 3.13 (1.66, 5.91) | 0.0004 | 0.99 (0.30, 3.21) | 0.982 | 1.03 (0.13, 7.89) | 0.977 |
| Chronic lung disease | 0.60 (0.27, 1.31) | 0.199 | 1.46 (0.67, 3.20) | 0.340 | 2.52 (0.72, 8.82) | 0.150 |
| Sepsis | 1.20 (0.48, 2.99) | 0.699 | 1.26 (0.30, 5.27) | 0.749 | - |  |
| Hypoxic spell | 1.09 (0.27, 4.47) | 0.902 | 2.07 (0.50, 8.64) | 0.317 | 19.13 (4.08, 89.60) | 0.0002 |
| Congestive heart failure | 1.15 (0.46, 2.86) | 0.769 | 2.54 (0.61, 10.65) | 0.201 | - |  |
| Ventilator support | 1.43 (0.87, 2.34) | 0.154 | 2.24 (1.17, 4.27) | 0.014 | 9.90 (2.71, 36.19) | 0.0005 |
| Complex heart (ref = TOF) | 1.01 (0.29, 3.50) | 0.990 | 5.59 (1.90, 16.41) | 0.002 | 2.85 (0.34, 23.61) | 0.332 |
| Single ventricle (ref = TOF) | 1.83 (0.72, 4.68) | 0.206 | 2.96 (1.24, 7.07) | 0.015 | 2.87 (0.83, 9.93) | 0.095 |
| PA-VSD (ref = TOF) | 1.50 (0.55, 4.06) | 0.428 | 1.33 (0.39, 4.56) | 0.646 | 1.50 (0.47, 4.74) | 0.492 |
| Prostaglandin E1 use | 1.58 (0.82, 3.02) | 0.172 | 2.35 (1.18, 4.66) | 0.015 | 4.35 (0.57, 33.35) | 0.157 |
| Times of having MBTS | 3.36 (0.82, 13.79) | 0.093 | 0.63 (0.09, 4.58) | 0.646 | 0.81 (0.32, 2.05) | 0.059 |
| Inotrope use 1 agent (ref=No) | 1.26 (0.64, 2.49) | 0.500 | 1.86 (0.94, 3.69) | 0.074 | 3.28 (1.06, 10.17) | 0.040 |
| Inotrope use > 1 agent (ref=No) | 4.32 (1.55, 12.03) | 0.005 | 11.21 (2.55, 49.23) | 0.001 | - |  |
| ASA classification 4 (ref=2 and 3) | 1.23 (0.94, 1.60) | 0.137 | 1.31 (0.93, 1.84) | 0.127 | 1.61 (0.91, 2.83) | 0.101 |
| Emergency case (ref=elective) | 0.79 (0.39, 1.61) | 0.519 | 1.69 (0.84, 3.41) | 0.143 | 6.38 (1.83, 22.25) | 0.004 |
| **Intraoperative period** |  |  |  |  |  |  |
| Inotrope use 1 agent (ref=No) | 0.83 (0.45, 1.54) | 0.562 | 1.70 (0.76, 3.78) | 0.194 | 1.10 (0.38, 3.18) | 0.857 |
| Inotrope use > 1 agent (ref=No) | 0.98 (0.49, 1.97) | 0.962 | 1.06 (0.35, 3.25) | 0.917 | 0.826 (0.10, 7.12) | 0.862 |
| Hemodilution | - |  | 1.16 (0.16, 8.51) | 0.884 | 1.13 (0.32, 3.94) | 0.850 |
| Shunt size/ weight ratio ≥0.65 (ref=<0.65) | - |  | 2.13 (0.89, 5.09) | 0.091 | 3.75 (1.22, 11.56) | 0.021 |
| Shunt size/ weight ratio ≥1.0 (ref=<1.0) | 4.20 (1.03, 17.19) | 0.046 | - |  | - |  |
| Hypoxemia with bradycardia (ref=No) | 1.35 (0.57, 3.21) | 0.499 | 1.88 (0.56, 6.31) | 0.306 | 15.03 (4.01, 50.27) | <0.0001 |
| Hypoxemia without bradycardia (ref=No) | 1.32 (0.77, 2.24) | 0.309 | 2.10 (1.05, 4.18) | 0.035 | 0.88 (0.20, 3.95) | 0.869 |
| Cardiac failure | 28.28 (7.63, 104.8) | <0.0001 | - |  | - |  |
| Cardiac arrest | 40.59 (12.12, 135.9) | <0.0001 | 4.82 (1.15, 20.18) | 0.031 | - | 0.975 |
| Blood loss (ml) | 1.011 (0.994, 1.029) | 0.220 | 1.006 (0.987, 1.026) | 0.519 | 0.998 (0.987, 1.009) | 0.724 |
| Duration of surgery (minutes) | 1.002 (0.994, 1.009) | 0.689 | 1.005 (0.993, 1.016) | 0.419 | 1.010 (0.996, 1.025) | 0.160 |
| **Postoperative period** |  |  |  |  |  |  |
| PostSpO_2_ – preSpO_2_ | 0.974 (0.948, 1.000) | 0.054 | 0.947 (0.925, 0.970) | <0.0001 | 0.960 (0.928, 0.993) | 0.017 |
| Duration of mechanical ventilator (days) | 1.001 (0.999, 1.002) | 0.067 | 1.000 (0.998, 1.002) | 0.857 | 0.999 (0.993, 1.005) | 0.803 |
| Length of ICU stay (days) | 1.012 (0.999, 1.026) | 0.074 | 0.995 (0.975, 1.015) | 0.634 | 0.967 (0.847, 1.103) | 0.616 |
| Length of hospital stay (days) | 1.003 (0.992, 1.014) | 0.598 | 0.995 (0.978, 1.012) | 0.587 | 0.998 (0.961, 1.040) | 0.910 |
| Shunt thrombosis | 2.93 (1.69, 5.09) | 0.0001 | 2.01 (0.84, 4.85) | 0.119 | 6.50 (2.10, 20.11) | 0.001 |
| Bleeding | 3.59 (2.00, 6.44) | <0.0001 | 4.41 (1.82, 10.66) | 0.001 | 5.01 (1.13, 22.22) | 0.034 |
| Pneumothorax | 1.45 (0.83, 2.52) | 0.194 | 1.19 (0.29, 4.98) | 0.807 | - |  |
| Pneumonia | 1.27 (0.77, 2.11) | 0.355 | 1.26 (0.63, 2.49) | 0.515 | 0.83 (0.19, 3.64) | 0.805 |
| Renal failure | 8.03 (3.03, 21.27) | <0.0001 | 5.02 (1.74, 14.43) | 0.003 | - |  |
| Sepsis | 2.71 (1.62, 4.54) | 0.0001 | 3.19 (1.54, 6.61) | 0.387 | 7.52 (2.10, 27.03) | 0.002 |
| Chylothorax/ perigraft seroma | 0.56 (0.14, 2.31) | 0.426 | 0.19 (0.03, 1.41) | 0.105 | 0.70 (0.09, 5.29) | 0.731 |
| Shunt revision | 2.25 (1.28, 3.97) | 0.005 | 1.07 (0.33, 3.52) | 0.906 | 1.72 (0.23, 13.14) | 0.600 |
| Numbers of reoperative thoracotomy during admission | 1.57 (1.15, 2.14) | 0.004 | 1.12 (0.72, 1.73) | 0.628 | 2.52 (1.16, 5.49) | 0.020 |
| Readmission within 30 day (ref=No) | 1.16 (0.28, 4.80) | 0.839 | 4.83 (0.64, 36.26) | 0.125 | - |  |
| Admission >30 days (ref=No) | 1.37 (0.81, 2.33) | 0.239 | 1.47 (0.71, 3.06) | 0.297 | 1.32 (0.17,10.05) | 0.786 |
| Admission >90 days (ref=No) | 2.19 (0.68, 7.02) | 0.189 | - |  | - |  |
| Total times of MBTS and other surgeries related to MBTS in all admission | 0.94 (0.66, 1.35) | 0.741 | 0.69 (0.41, 1.16) | 0.162 | 1.15 (0.66, 1.99) | 0.625 |

*by Wald test, ASA= American Society of Anesthesiologists, TOF=tetralogy of fallot, HR= hazard ratio, CI= confidence interval, MBTS=modified Blalock-Taussig shunt, PostSpO_2_=postoperative oxygen saturation, preSpO_2_=preoperative oxygen saturation, ICU=intensive care unit, PA-VSD=pulmonary atresia with ventricular septal defect.
