# Supplementary material for: Predictors of death after receiving a modified Blalock-Taussig shunt in cyanotic heart children: A competing risk analysis
Source: PLoS One. 2021 Jan 22;16(1):e0245754. doi: 10.1371/journal.pone.0245754 (PMC7822344; doi:10.1371/journal.pone.0245754)
Supplement: S3 Table — * p values were calculated using the Cox-proportional hazard model (Wald test), HR, hazard ratio; CI, confidence interval; MBTS, modified Blalock-Taussig shunt; PostSpO2, postoperative oxygen saturation; PreSpO2, preoperative oxygen saturation; TOF, tetralogy of fallot; PA-VSD, pulmonary atresia with ventricular septal defect. (DOCX) [file pone.0245754.s010.docx]

**S3 Table. Subgroup analysis predictors for time to death in children aged ≤1 month, and aged >1 month by time dependent multivariate Cox proportional hazard model (N=380).**

| **Variables** | **Aged ≤1 months (n=142, death 64)**  **Adjusted HR  (95% CI)** | **p value*** | **Aged >1-12 months (n=109, death 38)**  **Adjusted HR (95% CI)** | **p value*** | **Aged >12 months (n=129, death 17)**  **Adjusted HR  (95% CI)** | **p value*** |
| --- | --- | --- | --- | --- | --- | --- |
| **Preoperative** **period** |  |  |  |  |  |  |
| History of prematurity | - |  | 2.63 (1.08, 6.40) | 0.033 | - |  |
| Dextrocardia | 3.94 (1.97, 7.90) | 0.0001 | - |  | - |  |
| Complex heart (ref = TOF) | - |  | 6.80 (1.95, 23.72) | 0.003 | 6.00 (0.65, 55.09) | 0.113 |
| Single ventricle (ref = TOF) | - |  | 3.32 (1.30, 8.52) | 0.012 | 1.91 (0.41, 8.95) | 0.412 |
| PA-VSD | - |  | 0.68 (0.17, 2.76) | 0.589 | 0.59 (0.15, 2.30) | 0.449 |
| Inotrope use 1 agent (ref=no) | 1.58 (0. 96, 4.06) | 0.063 | 1.94 (0.85, 4.43) | 0.116 | - |  |
| Inotrope use >1 agent (ref=no) | 16.01 (4.87, 52.64) | <0.0001 | 11.23 (2.02, 62.55) | 0.006 | - |  |
| Emergency surgery (ref=elective) | - |  | - |  | 6.56 (1.50, 23.63) | 0.012 |
| **Intraoperative period** |  |  |  |  |  |  |
| Shunt size/weight ratio ≥0.65 (ref=<0.65) | - |  | 0.85 (0.32, 2.56) | 0.751 | 7.79 (1.74, 34.81) | 0.007 |
| Shunt size/weight ratio ≥1.0 (ref=<1.0) | 13.09 (2.79, 61.39) | 0.001 | - |  | - |  |
| Cardiac arrest | 17.66 (4.44, 70.18) | <0.0001 | 9.56 (0.89, 103.35) | 0.063 | - |  |
| Hypoxemia with bradycardia (ref=no) | - |  | - |  | 8.72 (1.11, 68.66) | 0.040 |
| Hypoxemia without bradycardia (ref=no) | - |  | - |  | 1.57 (0.32, 7.65) | 0.577 |
| **Postoperative period** |  |  |  |  |  |  |
| PostSpO_2_ – preSpO_2_ | 0.96 (0.93, 0.99) | 0.006 | 0.967 (0.94, 0.99) | 0.009 | 0.94 (0.89, 0.98) | 0.007 |
| Shunt thrombosis | 3.32 (1.84, 5.98) | <0.0001 | - |  | 4.50 (1.11, 18.26) | 0.035 |
| Bleeding | - |  | 5.29 (1.88, 14.85) | 0.002 | - |  |
| Sepsis | 2.88 (1.55, 5.35) | 0.0008 | 4.91 (1.98, 12.20) | 0.0006 | 17.92 (3.59, 89.52) | 0.0004 |

* p values were calculated using the Cox-proportional hazard model (Wald test), HR, hazard ratio; CI, confidence interval; MBTS, modified Blalock-Taussig shunt; PostSpO_2_, postoperative oxygen saturation; PreSpO_2_, preoperative oxygen saturation; TOF, tetralogy of fallot; PA-VSD, pulmonary atresia with ventricular septal defect.
